# Supporting SURgery with GEriatric Co-Management and AI (SURGE-Ahead): A study protocol for the development of a digital geriatrician

**DOI:** 10.1371/journal.pone.0287230

**Published:** 2023-06-16

**Authors:** Christoph Leinert, Marina Fotteler, Thomas Derya Kocar, Dhayana Dallmeier, Hans A. Kestler, Dennis Wolf, Florian Gebhard, Adriane Uihlein, Florian Steger, Reinhold Kilian, Annabel S. Mueller-Stierlin, Christoph W. Michalski, André Mihaljevic, Christian Bolenz, Friedemann Zengerling, Elena Leinert, Sabine Schütze, Thomas K. Hoffmann, Graziano Onder, Karen Andersen-Ranberg, Desmond O’Neill, Martin Wehling, Johannes Schobel, Walter Swoboda, Michael Denkinger, for the SURGE-Ahead Study Group

**Affiliations:** 1 Institute for Geriatric Research at AGAPLESION Bethesda Ulm, Ulm University Medical Center, Ulm, Germany; 2 Geriatric Center Ulm, Ulm, Germany; 3 DigiHealth Institute, Neu-Ulm University of Applied Sciences, Neu-Ulm, Germany; 4 Department of Epidemiology, Boston University School of Public Health, Boston, MA, United States of America; 5 Institute for Medical Systems Biology, Ulm University, Ulm, Germany; 6 Department for Orthopedic Trauma, Ulm University Medical Center, Ulm, Germany; 7 Institute of the History, Philosophy and Ethics of Medicine, Ulm University, Ulm, Germany; 8 Department of Psychiatry and Psychiatry II, Section of Health Economics and Health Services Research, Ulm University, Guenzburg, Germany; 9 Institute for Epidemiology and Medical Biometry, Ulm University, Ulm, Germany; 10 Department of General and Visceral Surgery, Ulm University Hospital, Ulm, Germany; 11 Department of Urology, Ulm University Hospital, Ulm, Germany; 12 Department of Gynecology and Obstetrics, Ulm University Hospital, Ulm, Germany; 13 Department of Otorhinolaryngology, Head and Neck Surgery, Ulm University Hospital, Ulm, Germany; 14 Department of Cardiovascular, Endocrine-metabolic Diseases and Aging, Instituto Superiore di Sanità, Rome, Italy; 15 Geriatric Research Unit, Department of Clinical Research, Faculty of Health, University of Southern Denmark, Odense, Denmark; 16 Centre for Ageing, Neuroscience and the Humanities, Trinity College Dublin, Dublin, Ireland; 17 Working Group FORTA, Faculty of Medicine Mannheim, Ruprecht-Karls-University of Heidelberg, Mannheim, Germany; PLoS ONE, UNITED STATES

## Abstract

**Introduction:**

Geriatric co-management is known to improve treatment of older adults in various clinical settings, however, widespread application of the concept is limited due to restricted resources. Digitalization may offer options to overcome these shortages by providing structured, relevant information and decision support tools for medical professionals. We present the SURGE-Ahead project (Supporting SURgery with GEriatric co-management and Artificial Intelligence) addressing this challenge.

**Methods:**

A digital application with a dashboard-style user interface will be developed, displaying 1) evidence-based recommendations for geriatric co-management and 2) artificial intelligence-enhanced suggestions for continuity of care (COC) decisions. The development and implementation of the SURGE-Ahead application (SAA) will follow the Medical research council framework for complex medical interventions. In the development phase a minimum geriatric data set (MGDS) will be defined that combines parametrized information from the hospital information system with a concise assessment battery and sensor data. Two literature reviews will be conducted to create an evidence base for co-management and COC suggestions that will be used to display guideline-compliant recommendations. Principles of machine learning will be used for further data processing and COC proposals for the postoperative course. In an observational and AI-development study, data will be collected in three surgical departments of a University Hospital (trauma surgery, general and visceral surgery, urology) for AI-training, feasibility testing of the MGDS and identification of co-management needs. Usability will be tested in a workshop with potential users. During a subsequent project phase, the SAA will be tested and evaluated in clinical routine, allowing its further improvement through an iterative process.

**Discussion:**

The outline offers insights into a novel and comprehensive project that combines geriatric co-management with digital support tools to improve inpatient surgical care and continuity of care of older adults.

**Trial registration:**

German clinical trials registry (Deutsches Register für klinische Studien, DRKS00030684), registered on 21^st^ November 2022.

## Introduction

Geriatric co-management is defined as the process of shared responsibility and decision making between a medical doctor, e.g. a surgeon, and a geriatrician or a multidisciplinary team aiming to prevent or treat geriatric complications [1]. It has reduced time-to-surgery, hospital and long term mortality, length of stay, and complications in a variety of health care settings across the globe [1–6]. Research has shown positive implications for different disciplines, such as trauma surgery [4], general surgery [7], oncological surgery [8], urology [9], and gynecology [10]. Despite these advantages, restricted financial resources and a limited number of expert geriatricians and their multidisciplinary teams make a widespread implementation of geriatric co-management in hospitals challenging [11, 12].

A key aspect of geriatric co-management is a comprehensive geriatric assessment (CGA) conducted by a multidisciplinary team. A CGA is a holistic approach for assessing a patient in different domains to determine physical, psychological, social, and functional impairments. Patients that underwent a CGA in the context of geriatric and surgical care are more likely to survive and less likely to be institutionalized [13, 14]. Another important aspect of geriatric co-management is individualized continuity of care (COC) planning. In complex health care systems, knowledge gaps concerning different options of continuous care for older people can lead to misdirection of patients. High level evidence indicates that a targeted discharge management reduces readmission rates, hospital length of stay, costs, and increases patient satisfaction [15]. In contrast, deficits in coordination of care transitions between different health care institutions have been identified as one aspect of iatrogenic disability [16].

In Germany, the amount of geriatric cases has doubled since 1999 and geriatric medicine has been the fastest growing clinical discipline in recent years [17]. Prospectively collected data from the German fragility fracture registry confirmed the benefit of orthogeriatric co-management on mortality [18]. Still, not all patients in need for geriatric care are treated accordingly. For example, in the federal state of Baden-Wuerttemberg less than 5% of geriatric patients, defined by multidimensional screening (“Geriatrie Check BW”), received a geriatric assessment in general practice. Only 22%, 38%, and 2% of geriatric patients with a stroke, femoral fracture, or heart failure, respectively, received treatment in either a department for geriatric rehabilitation or early inpatient geriatric rehabilitation [19].

Structures of geriatric care and options for the continuous care of older patients differ between federal states and regions [20]. Acute medical treatment with the option of early inpatient rehabilitation, day clinic medical treatment, and inpatient or outpatient geriatric rehabilitation can be available as individual or combined services. In Baden-Wuerttemberg, the main destinations after discharge of older people are inpatient acute geriatric care, inpatient acute care in another specialty, a geriatric or a specialized orthopedic or neurologic inpatient or outpatient rehabilitation, day care facilities, long term care facilities including nursing homes, or home with or without additional care.

Orthogeriatric co-management was announced to become a legal requirement for the treatment of older hip fracture patients in Germany by 2025 [21], increasing demand for geriatric expertise. Digital health interventions could help meet this demand [22]. To date, in geriatric medicine, digital technologies have been used to enhance patient assessment, e.g. via sensor-based gait analysis [23], continuous mobility monitoring [24], or digital patient-reported outcome measures for frailty assessment [25]. In the United Kingdom the identification of patients in need of geriatric services is possible using an automated hospital frailty risk score based on health administrative data on medical diagnosis [26]. Processing, analysis, and integration of these new data sources into existing structures is still lacking but has the potential to change common practice [27, 28].

By developing a digital health application, SURGE-Ahead (Supporting SURgery with GEriatric Co-Management and Artificial Intelligence) aims to bring geriatric expertise and support directly to the point of care in surgical departments in an effective and sustainable way.

## Methods

**Fig 1:**
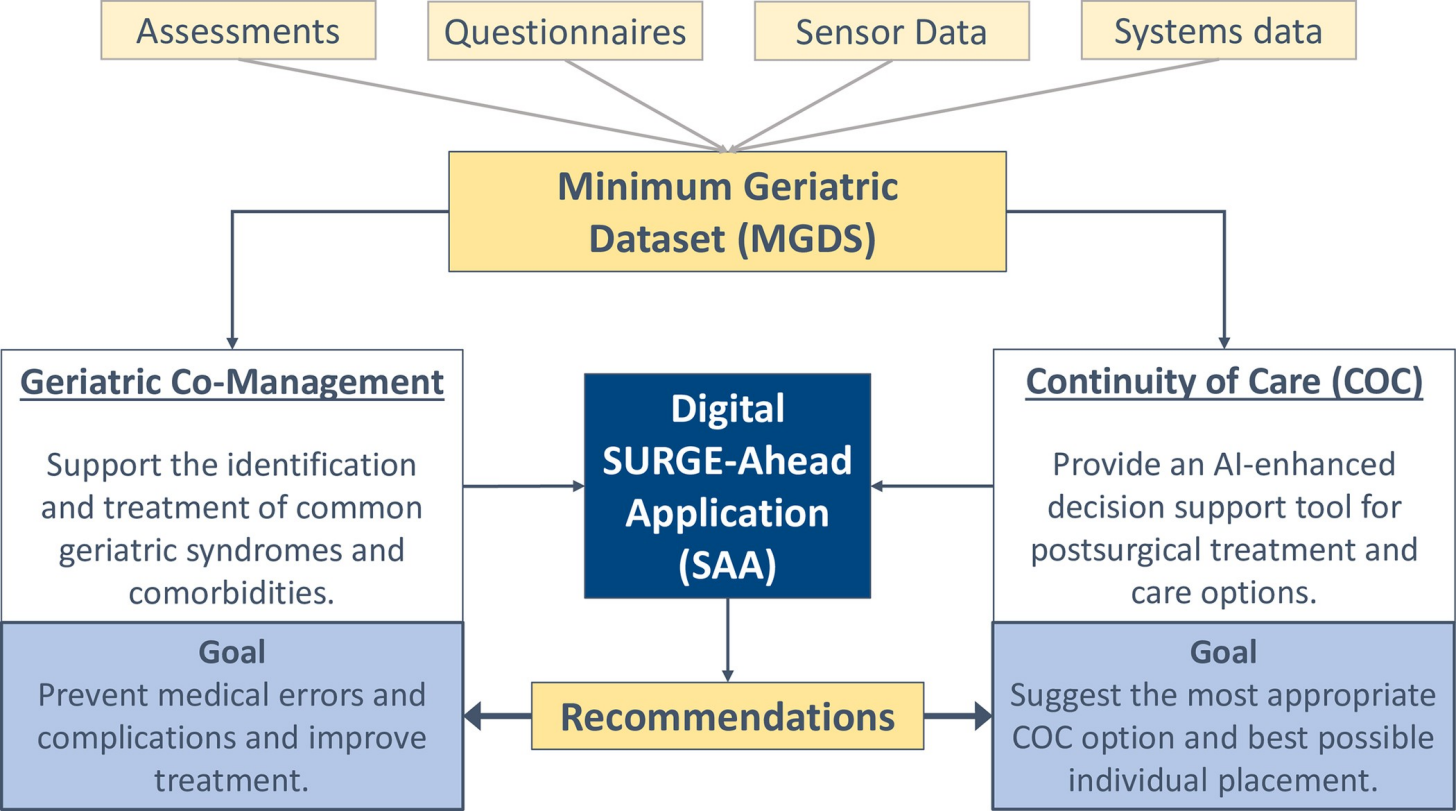
Core components of the SURGE-Ahead intervention. The figure shows the data sources of the minimum geriatric data set (MGDS) describing support of geriatric co-management and continuity of care as the two core components. After processing this input, the SURGE-Ahead application (SAA) will display recommendations for these components as data output.

The project aims to develop the SURGE-Ahead application (SAA, a dashboard-style user-interface) connected to the hospital information system (HIS) and the laboratory information system (LIS) that will display two core components: (1) evidence-based, easy-to-use clinical co-management recommendations and (2) artificial intelligence (AI)-enhanced continuity of care (COC) proposals. Functionality of these two core components will be based on a minimum geriatric dataset (MGDS) consisting of data from different sources (Fig 1).

The development, implementation, testing, and evaluation of the SURGE-Ahead application is a complex health care intervention. Therefore, SURGE-Ahead is structured according to the guidelines of the Medical Research Council (MRC) for development and evaluation of complex interventions [29, 30]. This development process encompasses four non-linear steps: Development, feasibility/piloting, implementation, and evaluation, with iterative improvements as the project progresses.

The current project focuses on the development of the SAA and will also prepare the implementation and piloting of the application. It is conducted in close cooperation with a multidisciplinary research team including geriatricians, computer scientists, surgeons, ethicists, epidemiologists, public health, and health economics specialists. Additionally, the team is supported by an international advisory board consisting of expert European geriatric researchers. The goals of the development phase are to establish an evidence base, develop a MGDS as input, and prepare evidence-based recommendations as output of the SAA. Furthermore, the digital application and its dashboard-style data user interface, data processing, AI integration, and connection to local hospital information system (HIS) and laboratory information system (LIS) will be programmed. The implementation of the SAA is prepared by analysis of local structures, stakeholder interviews, and functionality and usability tests with potential users. In a final step, AI training data will be gathered, co-management needs will be identified, and MGDS feasibility testing will be conducted in an observational and AI-development study. Ethical implications of the AI-enhanced digital health intervention will be constantly evaluated. The project has been reviewed in a peer-review- process and granted funding by the German Federal Ministry of Education and Research. Sponsor had no influence on study design, collection, management, analysis, or interpretation of data.

**Fig 2:**
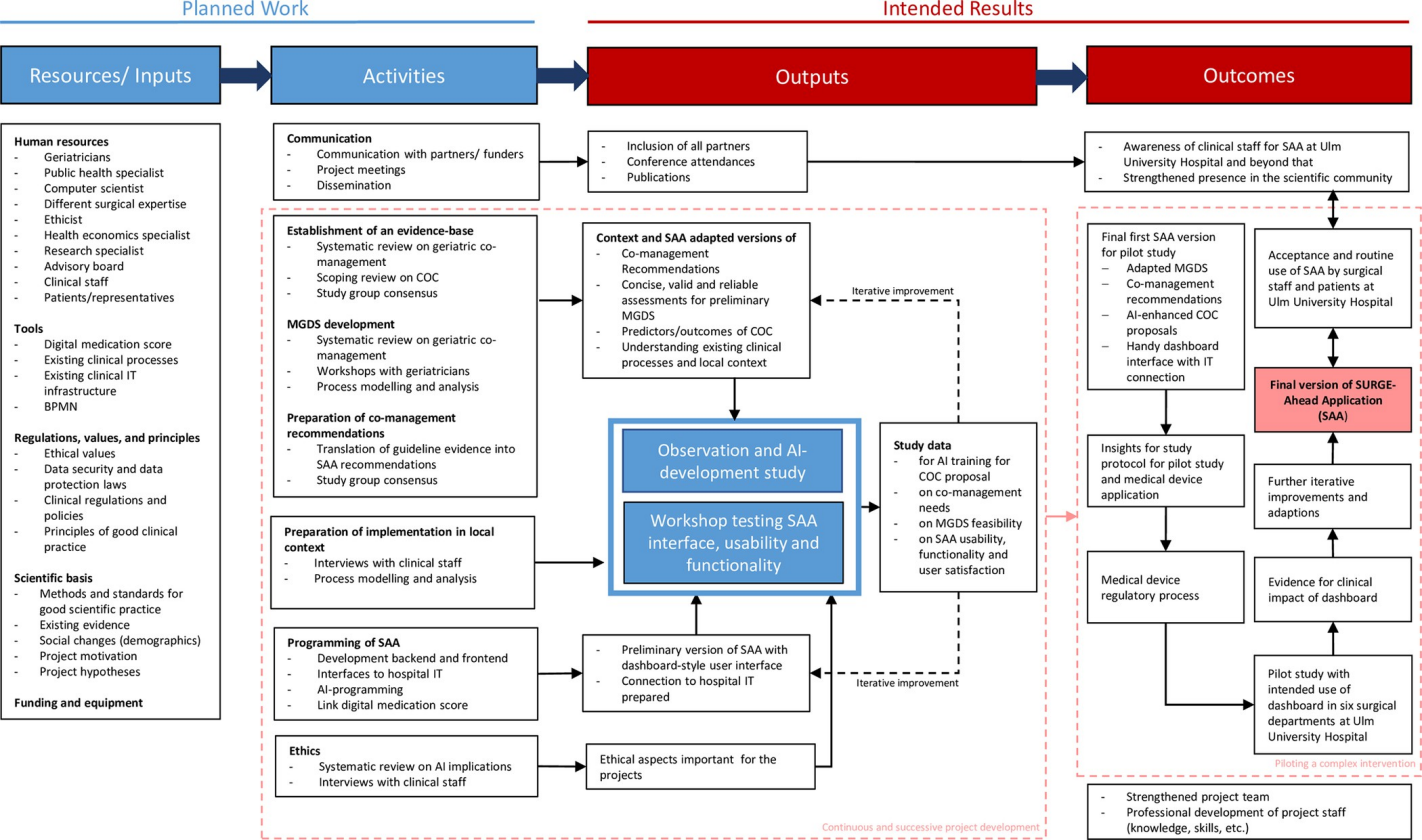
Logic model of the SURGE-Ahead project. The figure shows the different resources/inputs and activities as planned work and the outputs and outcomes as intended results of the project. Interactions between main components are highlighted. AI: artificial intelligence, BPMN: Business Process Model and Notation, COC: continuity of care, IT: information technology, MGDS: Minimum Geriatric Dara Set, SAA: SURGE-Ahead application.

Table 1 summarizes the main objectives of the SURGE-Ahead project and Fig 2 shows a logic model describing the development and piloting process.

**Table 1:** Main objectives of the SURGE-Ahead project.

Main objectives of the SURGE-Ahead project
1. Defining a minimum geriatric Data set (MGDS) as a comprehensive and concise assessment battery to be applied in the context of surgical care 2. Compiling practice-enhancing evidence-based suggestions for geriatric co-management 3. Programming an AI-enhanced decision support tool to improve continuity of care (COC) 4. Programming the digital SURGE-Ahead application (SAA) with a dashboard-style user interface connected to the hospital and laboratory information system that displays MGDS information as well as co-management and COC suggestions 5. Testing the usability and functionality of the SAA with potential users and stakeholders.

### Establishment of an evidence-base

Two major literature reviews, focusing on the two core components of the dashboard will be conducted.

#### Geriatric co-management

In a systematic review of clinical guidelines, current recommendations for geriatric co-management of orthogeriatric inpatients will be identified. The review will follow the Preferred Reporting Items for Systematic reviews and Meta-Analyses Extension for Scoping reviews (PRISMA-ScR) and a protocol has been published in the international prospective register of systematic reviews (PROSPERO) [31]. Only evidence-based guidelines published between 2016 and 2021 evaluating the level of evidence (e.g., by using the Grading of Recommendations, Assessment, Development and Evaluations (GRADE) framework) will be included. We chose to focus on orthogeriatric care because the highest-rated evidence in geriatric co-management exists in this field [1].

#### Continuity of care

The second core concept is the improvement of continuity of care decisions for older adults discharged from surgery. In a scoping review, eligible predictors and outcome measures of successful continuity of care decisions will be identified to be included in the MGDS and the AI algorithm used to propose the optimal discharge destination for each individual. As the topic and available literature is heterogenous including different study types, we decided to follow a scoping review approach. This methodology offers the opportunity for a broader view on a topic but supports only basic quantitative analysis [32]. The scoping review methodology of the Joanna Biggs Institute (JBI) and the PRISMA-ScR [33, 34] will be followed. A protocol has been published on Open Science Framework (OSF) [35].

### Development of the minimum geriatric data set (MGDS)

The MGDS is set up to be the comprehensive input data set of the study. It focuses on two aspects: (1) to provide enough detail to represent relevant aspects of a proper CGA conducted by a multidisciplinary team, (2) to be as concise and short as possible to be conducted in surgical settings with limited time and human resources.

Based on preliminary results from the literature review on geriatric co-management, the following topics and domains relevant to geriatric surgical care have been identified: Activities of daily living (ADL), frailty and multimorbidity, incontinence, mental health (delirium, dementia, depression), mobility and falls, nutrition, pain, polypharmacy, sensory impairment, and social support. These domains have been discussed with an international advisory board. Based on the utility of the assessments for clinical decisions, parsimony of data, and feasibility for the use in the context of a surgical department a first MGDS has been defined. In the observational study, the defined MGDS will be tested for feasibility and adapted, if necessary. The future SAA will be connected to existing IT-systems to include available data from the HIS and LIS (e.g., medications, social anamnesis, International Statistical Classification of Diseases and Related Health Problems (ICD) codes, laboratory results). In addition, we plan to include sensor-data, particularly for mobility and functionality parameters, provided by a six-axis accelerometer (AX6®, Axivity Ltd.). With the collected data, the final results of the conducted reviews, and the information and feedback provided by contributing surgeons and the advisory board the MGDS will be finalized.

### Preparation of co-management recommendations

On the basis of the systematic guideline review, evidence-based comprehensible co-management recommendations will be prepared for display as the output data of the SAA. The recommendations will cover identification and treatment of common geriatric syndromes like delirium, immobility or malnutrition. As an example, for patients with delirium or other cognitive impairments, resources and advice for non-pharmacological delirium prevention and management strategies should be provided to the treating medical teams [36, 37].

Furthermore, the SAA will incorporate an automated medication review plan to help manage polypharmacy and avoid potentially inappropriate medications (PIM). Optimizing medication in geriatric patients by deprescribing PIMs is an important aspect of geriatric co-management. Specifically, the reduction of anticholinergic medical burden is an integral part of multicomponent interventions to prevent delirium [38]. Not only over- but also undertreatment is a problem in older patients, e.g., specific osteoporosis treatment is often neglected. To address these issues the dashboard will include an analysis of the patient’s medication based on the Fit for The Aged (FORTA) classification of medication. This will be provided by the external, commercial partner OptiMedis AG® [39].

In addition, data collected during the pilot observational study will help to identify further needs for co-management in the local context, leading to an iterative improvement process. In a final step, the recommendations will be consolidated and approved by the scientific advisory board. The collected recommendations will be prepared for display in the digital dashboard.

### Programming of the digital SURGE-Ahead application (SAA)

#### Dashboard-style user interface

The front-end of the application will be based on the established Grafana framework, an open-source platform-independent application that allows the visualization of multiple data sources [40]. All graphical dashboard elements in Grafana are independent widgets predefined to display single values or time series such as the patient’s age or change of assessment ratings over time, respectively. Alternatively, Grafana can be extended by custom elements to add new functionalities such as data input. This way, clinical staff will be able to enter patient data (e.g., weight or assessment scores) directly into the dashboard. All data will be stored in a secure Redis database run within the hospital network and visualized in the dashboard applying evidence-based recommendations and rules.

#### AI for COC-recommendation

AI development follows the World Health Organization guidelines on ethics and governance of artificial intelligence for health [41]. Based on the data collected in the observation and AI-development study, multiple machine learning models of variant complexity will be implemented using languages such as R, Python, and Julia [42]. Both the expert recommendation of a geriatrician before discharge and follow-up data regarding COC quality will be evaluated as ground truth. A service will include the best-performing model and monitor incoming patient data to create or update COC-proposals. All new data will be used to retrain the model and constantly improve proposal accuracy. The model uncertainty and feature importance (i.e., parameters affecting the decision) will be displayed alongside each recommendation to improve model explainability and trust with the goal of a trustworthy AI according to the European Commission guidelines [43, 44].

#### Linkage to HIS and LIS

Data relevant for the MGDS will be identified in the local HIS and LIS and an automated extraction from IT-systems will be prepared for the final SAA.

### Preparation of implementation in local context

The departments for trauma surgery, general and visceral surgery, and urology of Ulm University Hospital will participate as the sites of primary implementation. As trauma surgery has the greatest need for geriatric co-management, a special focus will be put on this department. With the exception of the department of trauma surgery, where a liaison-based orthogeriatric co-management is already implemented, none of the other surgical departments have regular geriatric support.

Preparing the implementation of a complex intervention in the local context is an important step in the MRC framework. A lack of thorough preparation is a common reason why complex interventions do not transfer into clinical routine [45]. Local routines and personal preferences of stakeholders and clinical staff have to be incorporated into the development process. Several steps will be taken to ensure participation of local staff and the fit of the intervention to local structures.

Business Process Model and Notation (BPMN 2.0) will be used to model and analyze an exemplary process of an emergency femoral neck fracture admission and surgery. BPMN is a high-level standard to graphically represent the steps in a workflow and particularly useful for complex processes [46, 47]. Institutions or departments, participating personnel, necessary tasks, gateways, and resources will all be included in the BPMN-model, helping to identify the best options for the dashboard implementation.

Expert interviews with surgeons, nurses, social workers, and therapist will be conducted to identify local needs and resources concerning geriatric co-management of older patients. Additionally, personal attitudes regarding ethical implications of the project will be investigated to be incorporated during the dashboard development and implementation as needed.

To further incorporate the views of local stakeholders, consultant and attending surgeons of each department will be involved in the project development and participate in regular workshops and conferences to discuss and consent proceedings of the project with the study group and the international advisory board.

Reducing workload for clinical practitioners is an important requirement for a successful digital application. In this context, not only an efficient technical connection to existing IT systems, but also an intuitive and user-friendly interface, will be important for the success of the technology [48]. A prototype of the SAA will be evaluated and discussed with potential users of the application in a multi-stage, iterative process incorporating imaginary case examples as well as workshops. Usability, and functionality of the SAA will be evaluated with established tools such as the Technical Acceptance Model (TAM) or the Technology Usage Inventory (TUI) as well as self-designed feedback forms and improved accordingly [49, 50].

### Observation and AI-development study

#### Study design

A one-year prospective observational study with a 9-month recruitment and 3-month follow up period will be conducted in the departments of trauma surgery, general and visceral surgery, and urology starting in February 2023. The dashboard will not be displayed in the study, no intervention is planned. The main objectives are:

1. **Training of the AI**. Based on observational data a ground truth for AI training concerning COC proposals will be defined.
2. **Feasibility testing of MGDS**. The MGDS will be tested regarding feasibility and quality of data collection in a surgical setting.
3. **Analysis of a standard of care cohort**. The collected data will be used to identify specific local needs and resources for geriatric co-management.

#### Study population

Patients 70 years or older undergoing an emergency or elective surgery in the departments of trauma surgery, general and visceral surgery, or urology, an Identification of Seniors at Risk (ISAR)-Score of ≥2 and providing informed consent will be included [51]. In case of recruitment barriers patients with an ISAR-Score of ≥1 will be considered as well.

Informed consent will be obtained from all participants by study doctors. If consent is not possible because of cognitive decline, legal representatives will be contacted. Patients with a life expectancy of less than 3 month according to clinical judgement will be excluded. Patients can withdraw their participation at any timepoint. Reasons for discontinuation will be assessed if possible.

#### Sample size

Sample size is based on the minimum number of patients needed to train the AI algorithm [52]. Because higher numbers will improve AI performance a recruitment corridor between 170 to 240 patients including an expected dropout rate of 20% will be aimed for. Recruitment will focus on the department of trauma surgery, as this is where the greatest range and variability of possible COC options are covered. In addition, the majority of geriatric trauma patients are emergency cases with underlying geriatric conditions and are more likely to benefit from a subsequent co-management. The following target allocation has been determined: 120–190 participants in trauma surgery, 25 participants in general and visceral surgery, and 25 participants in urology.

#### Participants timeline

**Fig 3:**
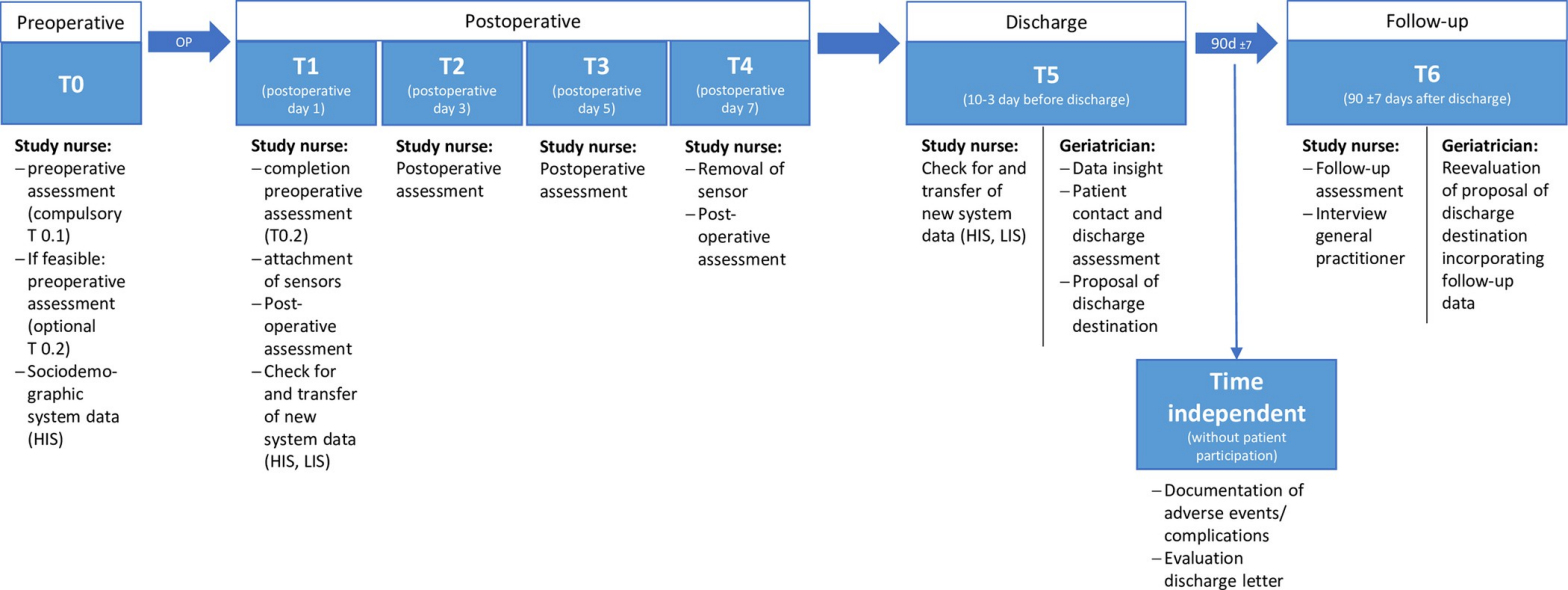
Participants timeline SURGE-Ahead observation and AI-development study. HIS: Hospital Information System, LIS: Laboratory Information System, SURG: Surgery.

Preoperative assessments (T0) are divided into compulsory preoperative (T0.1) and optional preoperative (T0.2) assessments. To acknowledge the potential lower resilience of some participants, T0.2 assessments can either be collected pre- or postoperatively, including collateral history. The postoperative interviews and assessments will be performed on days 1, 3, 5 and 7 after surgery (T1-T4). From postoperative day 1 until day 7 the mobility sensor will be attached. If patients are discharged earlier, the postoperative assessments will only be performed until the day of discharge. One to three days before discharge, the discharge assessments will be performed and the expert COC proposal will be documented by a geriatrician. After discharge, final data from the hospital information system data will be recorded, especially adverse events and complications. The medical discharge report will be critically assessed in terms of completeness and quality (time independent) [53]. A telephone follow-up will be performed with participants and general practitioners to evaluate COC decisions on discharge from surgery 90 days (± 7 days) after discharge (T6). Participants’ timeline is displayed in Fig 3.

#### Data collection, management and monitoring

Data at baseline and follow-up visits will be collected via a self-programmed electronic case report form (eCRF) hosted within the hospital network. All assessors and study nurses will be trained in the assessments and eCRF usage and regular monitoring will be performed. 30 patients (10 from each department) will self-complete a paper-based activity diary to evaluate accuracy of sensor data algorithms. Trained scientific data managers will double-check collected data in the eCRF database and query inconsistencies directly with the study nurses. Because of this ongoing monitoring of data quality, a data monitoring committee will not be installed. All data will be stored in a pseudonymized fashion and filed for at least 10 years.

#### Measurements

**Fig 4:**
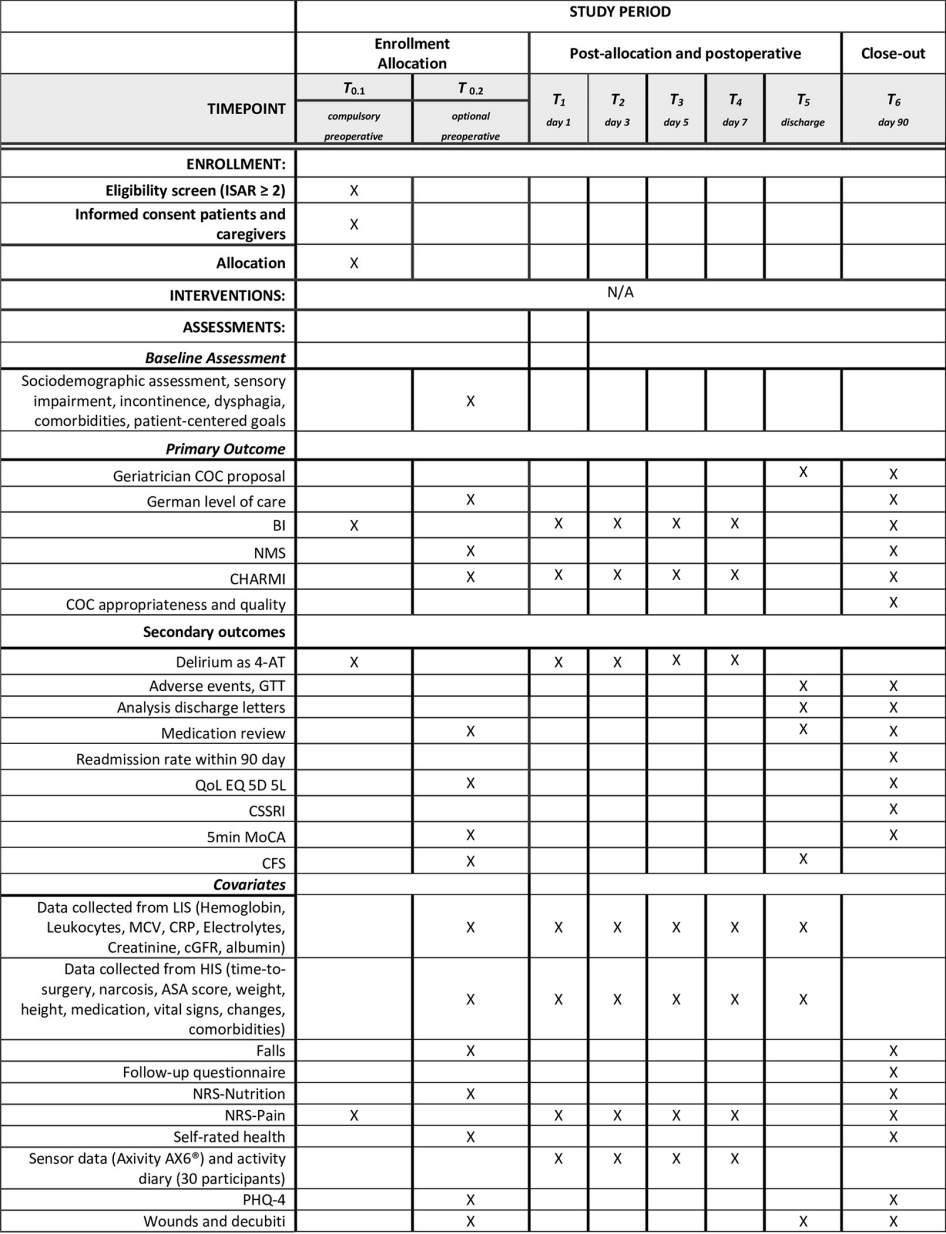
Schedule of enrollment and assessments for SURGE-Ahead observation and AI development study. 4-AT: 4 A´s test [57], 5min-MoCA: 5-min Montreal Cognitive Assessment [58], ASA: American Society of Anesthesiologists score, BI: Barthel Index [54], cGFR: calculated glomerular filtration rate with Cockroft Gault formular, CHARMI: Charité Mobility Index [56], CSSRI: Client Sociodemographic and Service Receipt Inventory [59], CFS: Clinical Frailty Scale [60], COC: continuity of care, CRP: c-reactive protein, EQ 5D-5L: EuroQoL 5D Health Questionnaire [61], GTT: Global Trigger Tool [62], HIS: hospital information system, LIS, Laboratory information system, MCV: mean cellular volume, NMS: New Mobility Score [55], NRS-pain: Numeric rating scale pain, NRS-nutrition: nutrition risk scale [63], PHQ-4: patient health questionnaire 4 items, QoL: Quality of Life.

Fig 4 shows all assessments and their schedule throughout the study phase.

Primary outcomes on level of care will be assessed in three ways:

1. An expert COC proposal by a geriatrician based on the collected MGDS data, the medical record and a personal patient contact will be made prospectively before discharge and reevaluated at 90 days follow up. This proposal will be used exclusively for the training of the AI and will have no influence on treatment and discharge decisions.
2. Any change of level of care (German: ‘Pflegegrad’), use of outpatient nursing services and institutionalization from baseline to discharge and 90 day follow.
3. Change from baseline to discharge and 90 days follow up of ADL and mobility measured by Barthel index (BI), New Mobility Score (NMS) and Charité Mobility Index (CHARMI) [54–56].

Self-reported opinion of participants and general practitioners on COC process will be documented at 90 days follow up to assess appropriateness of COC.

Secondary outcomes are listed in Fig 4.

#### Statistics

To identify determinants of COC proposals, regression analyses as well as machine learning approaches will be used. Descriptive statistical methods will be used to highlight specific local needs for geriatric co-management, e.g. delirium prevalence. MGDS feasibility and data quality will be evaluated by analysis of missing data patterns. Subgroup analysis will be done in participants enrolled in trauma surgery as well as for the documented discharge destinations. SAS, R, Python and Stata software will be used for statistical analyses and imputation. Health economic analyses will be conducted from the payer perspective by a preliminary cost-utility analysis with the net-monetary benefit regression approach [64–66]. Quality adjusted life years (QALY) will be estimated on the basis of the EQ-5D-5L using the current German value set [67–69].

#### Ethics approval and trial registration

This study received written ethical consent from the ethics committee of University of Ulm (# 310/22 dated 19th October 2022) (see S3 and S4 Files). Furthermore, the study has been registered in the German clinical trials registry (# DRKS00030684) on 21^st^ November 2022.

### Ethical aspects of AI-enhanced health interventions

An important point throughout the project is consideration of ethical concerns and challenges connected to the use of AI in medical care. The choice of suitable postsurgical treatment and care options is an important decision in surgical treatment and the responsibility of the treating consultant surgeon. Therefore, in the context of an AI-enhanced decision support tool, the proposals displayed by the dashboard have to be comprehensible and clearly marked as a suggestion not a decision.

Three requirements are indispensable for an adequate use of such AI-based support tool. Firstly, the treated patients need to be comprehensively informed about the use of such an application and potential risks of such use. The process of informed consent in case of AI-aided medical decision-making should encompass not only information disclosure but also understanding, voluntariness, and competence to decide [70]. Secondly, to comprehensively inform patients, physicians need to have relevant knowledge and understand implications of the use of this technology. Therefore, tailored training of clinical staff in the area of medical AI is required [70]. Thirdly, physicians using the application need to comprehend the rationale behind the AI-proposals. Use of “explainable AI” is recommended by the guidelines of the European Commission to increase trust and acceptance of the AI-generated suggestions for COC [43, 44, 71].

A systematic review concerning ethical aspects of AI-enhanced medical technologies will be conducted. Interviews concerning this topic with local clinical staff as well as patients and caregivers will be conducted, giving them the opportunity to express concerns but also potential chances they might see in the project. These findings will be integrated into the development of the SAA.

## Discussion

SURGE-Ahead aims do develop a complex digital health intervention, consisting of a digital version of a CGA presented in the user-friendly SAA that is connected to all relevant data sources and displays high-quality recommendations for geriatric co-management and COC decisions for geriatric patients undergoing a surgical procedure. According to the classification of digital health interventions of the World Health Organization, the programmed application can be categorized addressing clients, healthcare providers, and data services [22]. Before proceeding to a validation study of the dashboard intervention, our application will need to undergo the legal process of the European and German medical device regulation. In a second phase of the project, the clinical impact of the application will be tested at different surgical departments of Ulm University Hospital in a pilot interventional study. In addition to the departments of trauma surgery, general and visceral surgery, and urology, the recruitment is planned to be conducted in the departments of gynecology and ear-nose-throat (ENT) medicine.

The SURGE-Ahead intervention has the potential to change clinical practice. Although most current evidence on geriatric co-management focuses on orthogeriatrics, a CGA and geriatric co-management have also been beneficial in other surgical disciplines and non-emergency admissions [7–10]. In a recent study, CGA concepts did not work without a geriatrician and a multidisciplinary team, presumably because of the need for expert CGA interpretation and general guidance of the care process [74]. With a concise but comprehensive SAA we aim to partly overcome these issues by providing basic interpretation of MGDS data and AI-enhanced COC support. While for some geriatric patients, the automatically generated advice of the SAA might be sufficient to improve individual care, for others it might work as a differentiated screening tool for complex geriatric needs leading to a resource-oriented allocation of specific geriatric services [11]. While established screening tools have shown deficits in patient selection for geriatric involvement [72, 73], the intervention can offer a low-threshold service that highlights needs and might improve patient selection.

The implementation of geriatric health research knowledge into clinical practice is still insufficient, which has been described as the `know-do’ gap [74]. There are many reasons for this lack of implementation, including the multitude of specialized clinical disciplines treating geriatric patients and a lack of knowledge about geriatric medicine among health professionals in non-geriatric departments [75, 76]. The SAA can help to fill this `know-do’ gap by providing treatment teams with individualized geriatric information on real patient cases.

The SURGE-Ahead intervention does not attempt to replace geriatricians, but it should help to streamline provision of geriatric expertise in non-geriatric settings without initial involvement of a consultant geriatrician. Thereby, it should help to address upcoming shortages in geriatric capacities due to demographic changes [11].

All publications related to the SURGE-Ahead Project will be submitted to peer-reviewed national and international journals after approval from the project publication committee. Authorship eligibility criteria are set up by the publication committee and comply with International Committee of Medical Journal Editors (ICMJE) statements [77].

To conclude, SURGE-Ahead aims to sustainably implement a standardized, digital geriatric clinical decision support system in surgical departments to improve co-management and continuity of care of older patients admitted to these departments, especially in those settings where geriatric expertise is not easily available.

## Supporting information

S1 File Spirit checklist SURGE-Ahead study protocol. (PDF)

S2 File Funding confirmation SURGE-Ahead. (PDF)

S3 File Ethics committee approval SURGE-Ahead German original. (PDF)

S4 File Ethics committee approval SURGE Ahead English translation. (PDF)

S5 File Approved study protocol SURGE-Ahead German original. (PDF)

S6 File Approved study protocol SURGE-Ahead English translation. (PDF)
