# Supplementary material for: Supporting SURgery with GEriatric Co-Management and AI (SURGE-Ahead): A study protocol for the development of a digital geriatrician
Source: PLoS One. 2023 Jun 16;18(6):e0287230. doi: 10.1371/journal.pone.0287230 (PMC10275448; doi:10.1371/journal.pone.0287230)
Supplement: S2 File — (PDF) [file pone.0287230.s002.pdf]

BEAUFTRAGT VOM

Bundesministerium
für Bildung
und Forschung

DLR Projektträger

Deutsches Zentrum für Luft- und Raumfahrt e.V.

DLR Projektträger
Heinrich-Konen-Straße 1, 53227 Bonn

Herrn
Prof. Dr. Michael Denkinger
Universitätsklinikum
Institut für Geriatrische Forschung
Zollernring 26
89073 Ulm

DLR Projektträger
Bereich Gesundheit

Ihr Zeichen
Ihr Schreiben
Unser Zeichen

Ihr/e Gesprächspartner/in Dr. Michaela Fersch

Telefon +49 228 3821- 1268
Telefax +49 228 3821- 1257
E-Mail Michaela.Fersch@dlr.de

27. Januar 2023

To whom it may concern,

We hereby confirm that the project "SURGE-Ahead - Digitales Geriatrisches Co-Management mit Hilfe eines evidenz-basierten geriatrischen Assessments und künstlicher Intelligenz in der Chirurgie" (*engl. SURGE-Ahead - suporting surgery with geriatric co-management and artificial intelligence*; funding number: 01GY2101) is funded by the Federal Ministry of Education and Research (BMBF) within the funding programme "Strengthening Research in Geriatrics and Gerontology".

Further information of the project is accessible on the website:
https://www.gesundheitsforschung-bmbf.de/de/starkung-der-forschung-in-der-geriatrie-und-gerontologie-8925.php

Funded projects were all reviewed in a peer-review process.

Sincerely yours

i. A.

Dr. M. Fersch

Zertifiziert nach

Das Deutsche Zentrum für Luft- und Raumfahrt e. V. ist Mitglied der Helmholtz-Gemeinschaft. Vertreter des DLR sind der Vorstand und von ihm ermächtigte Personen. Auskünfte erteilt die Leitung Allgemeine Rechtsangelegenheiten, Linder Höhe, 51147 Köln (Hauptsitz des DLR).

Besucheradresse:
Heinrich-Konen-Straße 5
53227 Bonn
Telefon +49 228 3821-0
Internet DLR-PT.de
