# Supplementary material for: Supporting SURgery with GEriatric Co-Management and AI (SURGE-Ahead): A study protocol for the development of a digital geriatrician
Source: PLoS One. 2023 Jun 16;18(6):e0287230. doi: 10.1371/journal.pone.0287230 (PMC10275448; doi:10.1371/journal.pone.0287230)
Supplement: S3 File — (PDF) [file pone.0287230.s003.pdf]

universität ulm

Universität Ulm • Ethikkommission • 89069 Ulm

Ethikkommission

Vorsitz: Prof. Dr. Florian Steger

Geschäftsstelle: Iris Seitz

Hausadresse:
Helmholtzstraße 20 (Oberer Eselsberg)
89081 Ulm
Telefon: +49 – (0)731 – 500-22050/-33722
Telefax: +49 – (0)731 – 500-22036
Email: ethik-kommission@uni-ulm.de
http://www.uni-ulm.de/ethikkommission/

AGAPLESION BETHESDA ULM
Eing. 21. OKT. 2022
bearb.
erf. am
Kenntnis gen.

Herrn
Prof. Dr. med. Michael Denkinger
AGAPLESION BETHESDA KLIIK ULM
gemeinnützige GmbH
Akademisches Krankenhaus Universität Ulm
Zollernring 26
89073 Ulm

Unser Zeichen	Durchwahl	Datum
310/22 – Du/Sta	22050	19.10.2022

Antrag Nr. 310/22 – SURGE-Ahead Observations- und KI-Entwicklungsstudie (OKIE)

Sehr geehrter Herr Professor Denkinger,

Ihr o.g. Antrag lag der Ethikkommission der Universität Ulm am 05.09.2022 zur Beratung vor. Nach Eingang der gewünschten Ergänzungen und Korrekturen am 18.10.2022 sind nunmehr alle Voraussetzungen erfüllt.

Es bestehen keine ethisch begründbaren Bedenken gegen die Durchführung der geplanten Untersuchungen.

Damit wird die Bewertung durch die Ethikkommission der Universität Ulm mit einer zustimmenden Stellungnahme abgeschlossen.

Entsprechend der ausschließlich beratenden Funktion der Ethikkommission gemäß § 15 der Berufsordnung der Landesärztekammer Baden-Württemberg betrifft diese Bewertung nur die *berufsethische und berufsrechtliche Beurteilung* der Konzeption, der geplanten Methoden, der Durchführung und Überwachung des betreffenden Projekts sowie der beabsichtigten Patientenaufklärung.

Die ärztliche und juristische Verantwortung verbleibt uneingeschränkt beim Projektleiter und den daran Mitwirkenden.

- Mit der Rekrutierung von Probanden/Patienten darf erst ab Datum dieses Votums begonnen werden.
- Über alle schwerwiegenden oder unerwarteten unerwünschten Ereignisse, die während der Untersuchung auftreten und die Sicherheit der Studienteilnehmer oder die Durchführung des Projekts beeinträchtigen könnten, muss die Ethikkommission unverzüglich unterrichtet werden.
- Bitte geben Sie uns jede Änderung in der Protokolldurchführung an. Es muss dann geklärt werden, ob das Votum der Ethikkommission der Universität Ulm dann noch Bestand hat.
- Die Ethikkommission der Universität Ulm geht davon aus, dass nicht-ärztliche Mitarbeiter speziell auf Verschwiegenheit verpflichtet werden.
- Wir gehen davon aus, dass sämtliche studienbedingten Mehrkosten nicht den Kassen in Rechnung gestellt und bei der Rekrutierung von Studienteilnehmern keine dienstlichen oder andere Abhängigkeitsverhältnisse ausgenutzt werden.
- Wir weisen darauf hin, dass klinische Studien laut Art. 35 der Deklaration von Helsinki noch vor Rekrutierung der ersten Versuchsperson in einer öffentlich zugänglichen Datenbank (z.B. beim DRKS der Universität Freiburg) zu registrieren sind.
- Datenschutzrechtliche Aspekte von Forschungsvorhaben werden durch die Ethikkommission grundsätzlich nur kursorisch geprüft. Dieses Votum ersetzt mithin nicht die Konsultation des zuständigen Datenschutzbeauftragten.
- Außerdem benötigt die Ethikkommission eine zeitnahe Nachricht über den Abschluss der Studie und einen Bericht mit Mitteilung der gewonnenen Erkenntnisse.

Mitglieder der Kommission: Frau Prof. Dr. M. Dudeck, Prof. Dr. P. Gierschik, J. Glembek, Frau Dr. S. Hafner, Frau Prof. Dr. C. Herbert, Prof. Dr. J. Högel, Frau J. Kunecki, Prof. Dr. L.-H. Meyer, Frau Prof. Dr. B. Rack, Frau R. Rau, Prof. Dr. D. Rothenbacher, Prof. Dr. H. Schrezenmeier, Frau K. Staschelt, Prof. Dr. F. Steger, Prof. Dr. S. Stilgenbauer, PD Dr. M. Wittau

Für die Durchführung der Studie wünschen wir Ihnen viel Erfolg und bitten an dieser Stelle um Übersendung des Abschlussberichts bzw. der entsprechenden Publikation zu gegebener Zeit.

Für die Ethikkommission der Universität Ulm

Frau Prof. Dr. med. Manuela Dudeck
stellvertretende Vorsitzende

Anlage

Das Votum ergeht auf Grundlage folgender Dokumente:

Eingereichte Unterlagen [Eingang am 19.08.2022]:

- OKIE_Ethikantrag Ulm 17.08.2022, unterz. durch Prof. Denkinger, Prof. Gebhard, Prof. Michalski, Prof. Bolenz, Prof. Kestler, Prof. Kilian, Prof. Steger, Frau PD Dr. Dallmeier, Dr. Leinert, Frau Dr. Uihlein
- Begleit-Email Agaplesion Bethesda Klinik vom 18.08.2022 zur Antragseinreichung
- OKIE_Ausführliches Studienprotokoll August 2022
- OKIE_INFORMATION FÜR TEILNEHMENDE, Version Nr. 1, August 2022
- OKIE_INFORMATION FÜR GESETZLICHE VERTRETERINNEN UND VERTRETER, Version Nr. 1, August 2022
- OKIE_Einwilligungserklärung Teilnehmende, Version Nr. 1, August 2022
- OKIE_Einwilligung gesetzl. Vertreter:innen Version Nr. 1, August 2022
- OKIE_Erhebungsbogen mit allen Assessments und Fragen, Version 1 (August 2022)
- OKIE_MASKE FÜR DIE DATENERFASSUNG, Version Nr. 1, August 2022
- OKIE_Identifikationsliste_Studienteilnehmende
- OKIE_SCHWEIGEPFLICHTENTBINDUNG FÜR ANGEHÖRIGE UND HAUSÄRZT:INNEN DER TEILNEHMER:INNEN
- OKIE_SCHWEIGEPFLICHTENTBINDUNG FÜR BEZUGSPERSONEN UND HAUSÄRZT:INNEN DER TEILNEHMER:INNEN
- OKIE_Screening-Liste
- OKIE_Verschwiegenheitserklärung, Version Nr. 1, August 2022
- OKIE_Datenblatt_AX6
- OKIE_Hygienekonzept, Version Nr. 1, August 2022

Nachgereichte Unterlagen [Eingang am 18.10.2022]:

- Antwort Agaplesion Bethesda Ulm vom 13.10.2022 zu den Nachforderungen
- OKIE_Studienprotokoll_Revision, Version 2 vom Oktober 2022
- OKIE_Übersicht Promotionen
- Information für gesetzliche Vertreter/innen, Version 2 vom Oktober 2022
- Teilnehmerinformation, Version 2, Oktober 2022

Mitglieder der Kommission: Frau Prof. Dr. M. Dudeck, Prof. Dr. P. Gierschik, J. Glembek, Frau Dr. S. Hafner, Frau Prof. Dr. C. Herbert, Prof. Dr. J. Högel, Frau J. Kunecki, Prof. Dr. L.-H. Meyer, Frau Prof. Dr. B. Rack, Frau R. Rau, Prof. Dr. D. Rothenbacher, Prof. Dr. H. Schrezenmeier, [illegible]
