# Supplementary material for: Supporting SURgery with GEriatric Co-Management and AI (SURGE-Ahead): A study protocol for the development of a digital geriatrician
Source: PLoS One. 2023 Jun 16;18(6):e0287230. doi: 10.1371/journal.pone.0287230 (PMC10275448; doi:10.1371/journal.pone.0287230)
Supplement: S5 File — (PDF) [file pone.0287230.s005.pdf]

STUDIENPROTOKOLL

SURGE-Ahead Observations- und KI-Entwicklungsstudie (OKIE)

April 2023

Hiermit bestätige ich die Richtigkeit des Protokolls

________________________________ ________________________________

Datum, Ort

Unterschrift
Prof. Dr. Michael Denkinger, Studienleitung

Inhalt

Inhalt 2

1 Gesamtüberblick 4

1.1 Kernpunkte 4

1.2 Zusammenfassung 4

2 Verantwortlichkeiten 6

2.1 Beteiligte Institutionen 6

2.2 Studienleiter 6

2.3 Beteiligte Personen 6

2.4 Weitere Partner 8

3 Rationale 9

3.1 Hintergrund 9

3.2 Begründung für die durchzuführende Studie 10

3.3 Nutzen-Risiko-Abwägung 10

4 Studienziele 11

5 Studiendesign 12

5.1 Studiendesign 12

5.2 Studienpopulation 12

5.3 Fallzahlenschätzung 13

6 Studienablauf 14

6.1 Studiendauer 14

6.2 Vorbereitung der Studie 14

6.3 Rekrutierung der Proband:innen 14

6.4 Rekrutierung kognitiv eingeschränkter Proband:innen 15

6.6 Untersuchungszeitpunkte 17

7 Datenmanagement 19

7.1 Datensatz und Fragenkatalog 19

7.1.1 Existierende Datenquellen 21

7.1.2 Assessments und Fragebögen 22

7.1.3 Geriatrische Experteneinschätzung 22
7.1.4 Zeitunabhängiges Review der Patientenakte 22
7.1.5 Aktivitätsmessung mit dem Axivity AX6®-Sensor 23
7.2 Datenerfassung 24
7.3 Datenauswertung 25
7.3.1 Programmierung der KI für die Nachsorgeempfehlung 25
7.3.2 Deskription des aktuellen Behandlungsstandards 26
7.3.3 Vervollständigung des Datensatzes 26
7.3.4 Methodische Anpassungen 27
8 Unerwünschte Ereignisse (AE) / schwerwiegende unerwünschte Ereignisse (SAE) 28
8.1 (S)AE für Teilnehmende 28
8.2 Unerwünschte Ereignisse, die den Studienerfolg gefährden 28
9 Ethische und rechtliche Aspekte 30
9.1 Einwilligung 30
9.2 Kosten und Entschädigung für Teilnehmende 30
9.3 Risiken für Teilnehmende 30
9.4 Nutzen 31
9.5 Versicherung 31
9.6 Datenschutzkonzept 31
9.6.1 Pseudonymisierung der Studienteilnehmenden 31
9.6.2 Datenerfassung über die Eingabemaske 32
9.6.3 Datenerfassung durch die AX6®-Sensoren 32
9.6.4 Aufbewahrung und Archivierung der Daten 32
9.6.5 Datenschutz und Schweigepflicht 33
Literatur 34

1 Gesamtüberblick

1.1 Kernpunkte

- Prospektive Beobachtungsstudie über 12-15 Monate (9 Monate Rekrutierung (ggf. 12 Monate), 3 Monate Follow-up) an drei Kliniken des Universitätsklinikums Ulm (Klinik für Unfall-, Hand-, Plastische und Wiederherstellungschirurgie (UCH), Allgemein- und Viszeralchirurgie (AVC), Urologie und Kinderurologie (URO)).
- Evaluation physischer, funktioneller, sozialer und psychologischer Parameter, die für geriatrisches Co-Management und die Nachsorgeentscheidung im Rahmen der stationären Behandlung relevant sind.
- Nutzung der erhobenen Daten im weiteren Projektverlauf zur Programmierung einer künstlichen Intelligenz (KI), die einen Vorschlag für die bestmögliche Nachsorgeeinrichtung generiert und zur Analyse des aktuellen Behandlungsstandards.

1.2 Zusammenfassung

**Population**	Für einen operativen Eingriff stationär aufgenommene Patient:innen ≥ 70 Jahre mit einem ISAR[1]-Score ≥2
**Studiengröße**	N = 170 - 240 (inkl. 20% Drop-outs, 3 Kliniken: 120-190 UCH, 25 AVC, 25 URO)
**Studiendesign**	Prospektive Beobachtungsstudie
**Laufzeit**	12-15 Monate (9 Monate Rekrutierung (ggf. 12 Monate), 3 Monate Follow-up)<br>Start: 01.02.2023
**Ziel**	Erhebung eines Datensatzes<br>- für das Training der KI für die Nachsorgeempfehlung des Dashboards.<br>- als Vergleichskohorte für die im Verlauf (SURGE-Ahead Jahre 4-6) geplante Interventionsstudie (=Repräsentation des aktuellen Behandlungsstandards).
**Endpunkte**	<u>Primär:</u><br>Experteneinschätzung für die beste Nachsorgeoption bei Entlassung & Verifizierung bei Follow-up. Folgende Nachsorgeoptionen werden erfasst:<br>- Geriatrische Akutklinik<br>- Fachspezifische oder geriatrische Rehaklinik (stationär oder ambulant)<br>- nach Hause (mit oder ohne personelle/r Hilfe)<br>- Pflegeheim

[1] ISAR: Identifying Seniors at Risk (siehe auch Anhang Erhebungsbogen)

	<u>Sekundär:</u><br>- Selbstständigkeit in den Aktivitäten des täglichen Lebens und Pflegebedarf bei Entlassung und nach 3 Monaten<br>- Unerwünschte Ereignisse und Komplikationen während des stationären Aufenthaltes und in den ersten 90 Tagen nach Entlassung<br>- Medikamentenreview bei Entlassung und nach 3 Monaten<br>- Standardisierte Bewertung der medizinischen Entlassberichte<br>- Kognition während des stationären Aufenthaltes und nach 3 Monaten<br>- Mobilität während des stationären Aufenthaltes und nach 3 Monaten<br>- Wiedereinweisungsrate innerhalb von 3 Monaten nach Entlassung<br>- Lebensqualität der Teilnehmenden nach 3 Monaten<br>- Gesundheitsökonomische Betrachtung der in Anspruch genommenen Gesundheitsdienstleistungen bis drei Monate nach der Entlassung

2 Verantwortlichkeiten

2.1 Beteiligte Institutionen

- Institut für Geriatrische Forschung, Universitätsklinikum Ulm
- AGAPLESION Bethesda Klinik Ulm
- Institut für Medizinische Systembiologie, Universität Ulm
- Institut für Geschichte, Theorie und Ethik der Medizin, Universität Ulm
- Klinik für Psychiatrie und Psychotherapie II, Sektion Gesundheitsökonomie und Versorgungsforschung, Universitätsklinikum Ulm
- Klinik für Unfall-, Hand-, Plastische und Wiederherstellungschirurgie, Universitätsklinikum Ulm
- Klinik für Allgemein- und Viszeralchirurgie, Universitätsklinikum Ulm
- Klinik für Urologie und Kinderurologie, Universitätsklinikum Ulm

2.2 Studienleiter

Prof. Dr. Michael Denkinger

Ärztlicher Direktor, AGAPLESION Bethesda Klinik Ulm

Leitung Institut für Geriatrische Forschung, Universitätsklinikum Ulm

Mail: michael.denkinger@agaplesion.de, Tel: +49 (0)731 187-184

2.3 Beteiligte Personen

Institut für Geriatrische Forschung Universitätsklinikum Ulm und AGAPLESION Bethesda Klinik

- PD Ph.D. Dr. Dhayana Dallmeier: Leitung Forschungsabteilung AGAPLESION Bethesda Klinik Ulm
- Dr. Christoph Leinert: Oberarzt AGAPLESION Bethesda Klinik Ulm, Wissenschaftlicher Mitarbeiter Institut für Geriatrische Forschung
- Dr. Thomas Kocar: Facharzt AGAPLESION Bethesda Klinik Ulm, Wissenschaftlicher Mitarbeiter Institut für Geriatrische Forschung
- Marina Fotteler: Wissenschaftliche Mitarbeiterin
- Genia Decker: Medizinische Dokumentarin
- Gabriele Müller: Leitende Studienassistentin
- Cornelia Heth: Studienassistentin
- ~~N.N.: 2 Studentische Mitarbeitende zur weiteren Unterstützung der Datenerfassung~~

Institut für Medizinische Systembiologie, Universität Ulm

- Prof. Dr. Hans A. Kestler: Leitung Institut für Medizinische Systembiologie
- Dr. Dennis Wolf: Wissenschaftlicher Mitarbeiter

Institut für Geschichte, Theorie und Ethik der Medizin, Universität Ulm

- Prof. Dr. Florian Steger: Direktor Institut für Geschichte, Theorie und Ethik der Medizin
- Dr. Marcin Orzechowski: Wissenschaftlicher Mitarbeiter

Klinik für Psychiatrie und Psychotherapie II, Sektion Gesundheitsökonomie und Versorgungsforschung, Universitätsklinikum Ulm

- Prof. Dr. Reinhold Kilian: Leitung Sektion Gesundheitsökonomie und Versorgungsforschung
- Dr. Annabel S. Müller-Stierlin: Wissenschaftliche Mitarbeiterin

Klinik für Unfall-, Hand-, Plastische und Wiederherstellungschirurgie (UCH)

- Prof. Dr. Florian Gebhard: Ärztlicher Direktor
- PD Dr. Konrad Schütze: Oberarzt
- Dr. Adriane Uihlein: Fachärztin
- Dr. Raffael Cintean: Assistenzarzt
- Dr. Carlos Pankratz, Assistenzarzt
- Sibylle Beck: Studienassistentin
- Esther Blaum: Studienassistentin

Klinik für Allgemein- und Viszeralchirurgie (AVC)

- Prof. Dr. Christoph Michalski: Ärztlicher Direktor
- Prof. Dr. André Mihaljevic: Leitender Oberarzt
- Nadir Nasir: Assistenzarzt
- Vytautas Stasiunaitis: Assistenzarzt
- Colette Dörr-Harim: Leitung Studienzentrum
- Karen Clauss: Studienassistentin

Klinik für Urologie und Kinderurologie (URO)

- Prof. Dr. Christian Bolenz: Ärztlicher Direktor
- Dr. Felix Wezel: Leitender Oberarzt
- PD Dr. Friedemann Zengerling: Oberarzt
- Fabia Mangold: Assistenzärztin
- Vanessa Disque: Studienassistentin
- Carmen Veliz-Torrico: Studienassistentin

2.4 Weitere Partner

Optimedis AG, Hamburg

Pascal Wendel: Leiter Gesundheitsdatenanalyse und IT

Sektion Versorgungsforschung und Rehabilitationsforschung (SEVERA), Institut für Medizinische Biometrie und Statistik (IMBI), Universitätsklinikum Freiburg

Dr. Sebastian Voigt-Radloff: Leitung Forschungsbereich Versorgungsforschung mit Schwerpunkt Therapie- und Pflegewissenschaften

AOK Baden-Württemberg, Referat Versorgungsinnovation

Anna-Lena Flagmeier: Projektkoordination

Hochschule für angewandte Wissenschaften Neu-Ulm (HNU)

- Prof. Dr. Walter Swoboda, Forschungsprofessor und Leiter Institut DigiHealth
- Prof. Dr. Johannes Schobel, Forschungsprofessor Digitale Medizin und Pflege

3 Rationale

3.1 Hintergrund

Ein geriatrisches Co-Management chirurgischer Patient:innen kann die Behandlung verbessern, die Schwere von Langzeitfolgen verringern und die Mortalität reduzieren (1–3). Besonders erfolgreich war die Integration geriatrischer Expertise bisher in der Unfallchirurgie (4). Aber auch in anderen chirurgischen Fachbereichen, wie der Allgemeinchirurgie (5) oder der Urologie (6) können Patient:innen von geriatrischem Co-Management profitieren.

Ein Kernaspekt des geriatrischen Behandlungsansatzes ist die holistische Betrachtung der Patient:innen mittels eines comprehensive geriatric assessment (CGA), das von einem multidisziplinären Team durchgeführt wird. Bei einem CGA werden unterschiedliche Domänen betrachtet, um physische, psychologische, soziale und funktionelle Einschränkungen zu identifizieren und in die Behandlung miteinzubeziehen (7,8).

Angesichts der steigenden Anzahl geriatrischer PatientInnen und des Mangels an ausgebildeten Geriater:innen lässt sich ein geriatrisches Co-Management jedoch trotz der Vorteile bisher nicht flächendeckend etablieren. Eine besondere Herausforderung in der Betreuung geriatrischer Patient:innen stellt die Einschätzung des optimalen Nachsorgepfades dar (z.B. Entlassung nach Hause, in ein Pflegeheim, in eine geriatrische Rehaklinik oder in eine geriatrische Akutklinik). Im Projekt SURGE-Ahead soll eine digitale Anwendung (Dashboard) für die Verbesserung des geriatrischen Co-Managements in chirurgischen Klinken entwickelt werden. Anstelle eines CGA wird ein Datensatz definiert, der die Minimalanforderungen für erfolgreiches geriatrisches Co-Management abbildet (Minimum Geriatric Dataset – MGDS). Basierend auf dem MGDS soll das Dashboard Vorschläge zu 1) evidenzbasierten Behandlungsoptionen typischer geriatrischer Erkrankungen und Syndromen auf Basis einfacher Algorithmen und 2) Vorschläge für eine optimale Nachsorgeeinrichtung auf Basis einer künstlichen Intelligenz (KI) anzeigen. Mit dem Dashboard wird geriatrische Expertise in chirurgischen Kliniken zur Verfügung gestellt. Das System soll im Betrieb das gesamte, multidisziplinäre Team durch eine erste Einschätzung und Behandlungsempfehlungen unterstützen. Das Ziel ist eine nachhaltige Verbesserung von Behandlung und Weiterversorgung älterer PatientInnen.

SURGE-Ahead ist im Juli 2021 gestartet. Die ersten drei Projektjahre sind der Entwicklung des Dashboards gewidmet. Für den Betrieb des Dashboards wird, basierend auf Evidenz und Expertenkonsens, das MGDS definiert. Das MGDS setzt sich zusammen aus 1) prä- und postoperativ durchzuführenden Assessments und Fragebögen (siehe Kapitel 7.1), 2) bereits vorhandenen Daten aus dem Krankenhaus- und Laborinformationssystemen und 3) Daten zu Bewegungs- und Mobilitätsparametern, die über einen Körpersensor (Axivity AX6) erhoben werden.

3.2 Begründung für die durchzuführende Studie

In der geplanten Observations- und KI-Entwicklungsstudie (OKIE) soll in drei Kliniken des Universitätsklinikums Ulm (Unfall-, Hand-, Plastische und Wiederherstellungschirurgie, Allgemein- und Viszeralchirurgie, Urologie und Kinderurologie) mit 170-240 Patient:innen die Datenbasis für das Training und die Entwicklung der KI gesammelt werden. Mit diesem Baustein kann die Entwicklung des Dashboards im Anschluss finalisiert werden. Die OKIE dient damit zur Fertigstellung der Programmentwicklung und Vorbereitung einer Interventionsstudie in den darauffolgenden Jahren.

3.3 Nutzen-Risiko-Abwägung

Die durchzuführenden Assessments bedeuten einen Mehraufwand von knapp zwei Stunden für die Proband:innen. Diese Zeit wird aufgeteilt in präoperative und postoperative Assessments sowie ein ca. 30-minütiges telefonisch erhobenes Follow-up ca. 90 Tage nach der Entlassung. Die Gefahr, dass die Assessments die Risiken der aktuellen individuellen Krankenhausbehandlung der Patient:innen übersteigen, besteht nicht. Sollte die Befragung für die Patient:innen eine Belastung darstellen, wird diese umgehend unterbrochen und ggf. zu einem späteren Zeitpunkt fortgesetzt. Alle Teilnehmenden können jederzeit und ohne Angabe von Gründen von der Studie zurücktreten. Die Assessments werden durch geschulte Kräfte (Studienassistent:innen, Studienärzt:innen) durchgeführt. Während der stationären Studienphase stehen die Studienärzt:innen und -assistent:innen für Fragen zur Verfügung.

Auf Wunsch können die gewonnenen Erkenntnisse nach Abschluss der Studie an die Proband:innen weitergegeben werden. Darüber hinaus leisten die Proband:innen einen wertvollen Beitrag zum wissenschaftlichen Erkenntnisgewinn.

4 Studienziele

Im Rahmen der OKIE soll der für SURGE-Ahead definierte Datensatz (MGDS) für 170 bis 240 Patient:innen an den drei beteiligten Kliniken erhoben werden. Zusätzlich zum MGDS wird ein/e Geriatrie-erfahrene Ärzt:in vor der Entlassung aus dem Krankenhaus basierend auf den erhobenen MGDS Daten, der Krankenakte und einem persönlichen Patientenkontakt eine Empfehlung für eine Nachsorgeoption für alle Proband:innen dokumentieren. Diese Empfehlung wird beim Follow-up erneut verifiziert, ggfs. korrigiert und als Goldstandard festgelegt. Dieser dient dazu, die KI zu trainieren, die im finalen Dashboard einen Vorschlag für die bestmögliche Nachsorgeeinrichtung generieren soll.

Das primäre Ziel der OKIE ist demnach die Erhebung eines Datensatzes für das Training der KI, die eine Empfehlung für eine Nachsorgeeinrichtung generieren soll.

Als sekundäres Ziel wird angestrebt, den Datensatz der OKIE als Vergleichskohorte für die im Verlauf (SURGE-Ahead Jahr 4-6) geplante Interventionsstudie mit dem fertiggestellten Dashboard zu nutzen (=Repräsentation des aktuellen Behandlungsstandards).

5 Studiendesign

5.1 Studiendesign

Es wird eine **prospektive Beobachtungsstudie mit einem Follow-up nach drei Monaten** in den Kliniken für Unfall-, Hand-, Plastische und Wiederherstellungschirurgie, Allgemein- und Viszeralchirurgie und Urologie und Kinderurologie des Universitätsklinikums Ulm durchgeführt. Es erfolgt keine Intervention, während der Studie wird also nicht in die Behandlung eingegriffen.

Zu verschiedenen Assessmentzeitpunkten und beim Follow-up werden für die Behandlung und zur Beurteilung des Behandlungserfolges geriatrischer Patient:innen wichtige Daten erhoben (MGDS). Ein/e Geriatrie-erfahrene Ärzt:in aus dem Projektteam wird basierend auf den erhobenen MGDS Daten, der Krankenakte und einem persönlichen Patientenkontakt darüber hinaus für alle Proband:innen eine Experteneinschätzung für eine optimale Nachsorgeoption dokumentieren. Diese Einschätzung wird beim Follow-up nochmals durch dieselbe/denselben Geriatrie-erfahrene Ärzt:in verifiziert und dient anschließend als Referenzgröße für das Training der KI. Die Einschätzung der/des Geriatrie-erfahrenen Ärztin/Arztes wird dem behandelnden klinischen Personal nicht gezeigt und hat keinen Einfluss auf die Nachsorge der Proband:innen.

Dem Studiendesign entsprechend ist eine Verblindung des Studienteams nicht möglich und auch nicht erforderlich, da keine Intervention erfolgt.

5.2 Studienpopulation

<u>Einschlusskriterien</u>

- Patient:innen ≥ 70 Jahre, die für einen stationären Aufenthalt mit operativem Eingriff in einer der drei beteiligten Kliniken aufgenommen werden und deren Operation noch nicht durchgeführt wurde (Notfall- oder Elektivaufnahmen).
- Patient:innen mit einem ISAR-Score ≥ 2 (9,10).

<u>Ausschlusskriterien</u>

- Patient:innen mit einer palliativen Behandlungssituation (Lebenserwartung < 3 Monate basierend auf klinischer Einschätzung des behandelnden Arztes).
- Nicht-Einwilligungsfähige Patient:innen für die keine gesetzlichen Betreuer:innen oder bevollmächtigte Person vorhanden sind.
- Erhebung der Assessments aufgrund eingeschränkter Kommunikationsfähigkeit (z. B. wegen fehlender Sprachkenntnisse) nicht möglich.
- ~~Patient:innen mit einer bestätigten COVID-19-Infektion.~~
- Patient:innen, die bereits an einer anderen Studie teilnehmen.

- Patient:innen mit einer vermuteten Verweildauer von <3 Nächten.

5.3 Fallzahlenschätzung

Eine größere Anzahl von Datensätzen verbessert die Performance der zu entwickelnden KI. Ist der Datensatz zu klein besteht das Risiko, dass die KI nicht adäquat trainiert wird, sondern die Entscheidung größtenteils zufällig trifft (11). Ausgehend von einer Mindestanzahl wird daher ein Rekrutierungskorridor angestrebt, der bei gutem Rekrutierungsfortschritt ggf. ausgereizt werden kann. Mit einer erwarteten Dropout-Rate von 20% sollen 170 - 240 Patient:innen rekrutiert werden. Hierbei wird der Fokus auf die Klinik für Unfall-, Hand-, Plastische und Wiederherstellungschirurgie gelegt, da hier die größte Bandbreite und Variabilität der möglichen Nachsorgeoptionen abgedeckt wird und die hier behandelten Patient:innen voraussichtlich den größten Nutzen einer späteren Dashboard-Anwendung haben. Folgende Aufteilung wird angestrebt (kann im Verlauf der Studie leicht angepasst werden):

- UCH: 120 - 190 Proband:innen
- AVC: 25 Proband:innen
- URO: 25 Proband:innen

Ausgehend von ±1000/Jahr stationär behandelten Patient:innen ≥ 70 Jahren in der Klinik für Unfall-, Hand-, Plastische und Wiederherstellungschirurgie (wovon ±500/Jahr einen ISAR von 2 oder höher haben), ±800/Jahr in der Klinik für Urologie und Kinderurologie und ±1400/Jahr in der Klinik für Allgemein- und Viszeralchirurgie, erscheinen die Rekrutierungszahlen realistisch.

Die angestrebten Fallzahlen basieren auf einer Einschätzung der Machbarkeit bzgl. KI-Training und Rekrutierung, es erfolgte keine explizite Fallzahlkalkulation.

6 Studienablauf

6.1 Studiendauer

Die Studie ist geplant für 12 Monate vom 01.02.2023 bis zum 31.01.2024 mit einem Rekrutierungszeitraum von neun Monaten und einem Follow-up von drei Monaten. Sollte das Rekrutierungsziel innerhalb der neun Monate nicht erreicht werden, kann der Rekrutierungszeitraum um weitere drei Monate verlängert werden. Mit Follow-up läuft die Studie in diesem Fall bis maximal zum 30.04.2024.

6.2 Vorbereitung der Studie

Zwischen 01.12.2022 und 31.01.2023 wird die Studie vorbereitet. Die Studienassistent:innen, Ärzt:innen, das Pflegepersonal und die Datenmanagerin werden in die Studie und den Studienablauf eingewiesen. Pro Klinik wird mind. eine Informationsveranstaltung durchgeführt, bei der es auch die Möglichkeit gibt, Fragen zu stellen. Das Ziel ist, das klinische Personal vor Ort bestmöglich auf die anlaufende Studie vorzubereiten, um Unterstützung bei der Auswahl und Rekrutierung geeigneter Patient:innen zu erhalten.

Vor Ort in den Kliniken sind die Studienassistent:innen die Ansprechpartner für akute Fragen. Hier wird darauf geachtet, eine möglichst umfassende Präsenz zu gewährleisten. Darüber hinaus stehen in allen drei Kliniken ärztliche Ansprechpartner:innen zur Verfügung (siehe Kapitel 2.3), die auch Teil des SURGE-Ahead Studienteams sind.

6.3 Rekrutierung der Proband:innen

Das Screening mit dem ISAR-Score ist eine offizielle Empfehlung und in den Zentren bekannt. In den meisten Fällen wird der Score standardmäßig bei der Aufnahme erhoben. Für den Zweck der OKIE, werden alle Patient:innen ab 70 Jahre, die in UCH, AVC oder URO für einen operativen Eingriff stationäre aufgenommen werden mit dem ISAR gescreent. Die Rekrutierung der möglichen Proband:innen erfolgt nach Bestätigung der Einschlusskriterien. Die Prüfung der Einschlusskriterien erfolgt zugunsten eines einfacheren Patientenkontaktes papierbasiert. Bei erfolgtem Einschluss werden die Daten nachträglich elektronisch erfasst (siehe auch Kapitel 7.2).

Mögliche Proband:innen, werden unmittelbar nach der stationären Aufnahme oder, im Falle von Elektivoperationen, bei einer Vorbesprechung, über die Möglichkeit einer Studienteilnahme am SURGE-Ahead-Projekt informiert. Bei vorliegendem Interesse werden die Patient:innen von den Studienärzt:innen über Ziele, Abläufe, Risiken und Datenschutz des Forschungsvorhabens mündlich und schriftlich aufgeklärt und erhalten eine schriftliche Patient:inneninformation (siehe Anhang:

Patient:inneninformation). Teil der Aufklärung ist auch die Information über die Nutzung eines lumbal auf der Haut angebrachten Sensors (Fa. Axivity AX6®) zur Erhebung von postoperativen Mobilitätsdaten während des stationären Aufenthaltes.

Alle Patient:innen haben während der Aufklärung die Möglichkeit, Fragen zu stellen. Die Einwilligung zur Teilnahme an der Studie erfolgt in mündlicher und schriftlicher Form und ist freiwillig (siehe Anhang: Einwilligung).

Für den Fall, dass die Proband:innen im Rahmen der Befragungen auf Grund Ihrer Grunderkrankung (z.B. Fraktur) situativ zu sehr belastet sind und/oder aus anderen Gründen nicht in der Lage oder willens sind die Befragung fortzuführen, können sie diese jederzeit unterbrechen. Für diesen Fall besteht die Möglichkeit, dass einige Daten auch via Fremdanamnese von einer Bezugsperson (z.B. Angehörige, Freunde) erhoben werden.

Im Rahmen der Follow-up-Befragung (T6) soll zusätzlich auch die Hausärztin/der Hausarzt der Probandin/des Probanden bezüglich der Nachsorgequalität befragt werden (Fragen siehe Anhang: Erhebungsbogen). Um die Befragung von Angehörigen und Hausärzt:innen zu ermöglich erteilen die Proband:innen eine Schweigepflichtentbindung (siehe Anhang: Schweigepflichtentbindung). Die benötigten Kontaktdaten für Angehörige und Hausärzt:innen werden zunächst in der Schweigepflichtentbindung erfasst und später zusammen mit der Identifikationsnummer der Probandin/des Probanden in die papierbasierte Identifikationsliste übertragen (siehe Anhang: Identifikationsliste). Die Schweigepflichtentbindung wird bei Aufnahme in die Studie angefragt, kann aber auch im Verlauf der Studienteilnahme unterzeichnet werden. Ohne unterzeichnete Schweigepflichtentbindung kann jedoch keine Befragung der Hausärztin/des Hausarztes im Rahmen des Follow-ups durchgeführt werden.

Nach Einwilligung der Patientin/des Patienten wird die Patientin/der Patient in die Studie aufgenommen. Zur Dokumentation von Patient:innenauswahl und Rekrutierungsfortschritt wird in allen Zentren eine anonymisierte Screeningliste mit Angabe etwaiger Ausschlussgründe geführt (siehe Anhang: Screeningliste).

6.4 Rekrutierung kognitiv eingeschränkter Proband:innen

Gemäß der Deklaration von Helsinki in der Fassung vom Oktober 2013 (unter Berücksichtigung der Punkte 28.-30.) ist der Einschluss nicht-einwilligungsfähiger Menschen in medizinische Studien nur unter engen Voraussetzungen zulässig (12). Diese sehen vor, dass den Patient:innen (1) entweder ein direkter eigener Nutzen aus der Studienteilnahme erwächst, oder (2) die Gruppe von Patient:innen, die dieses Individuum repräsentiert durch die Erkenntnisse der Studie voraussichtlich einen Nutzen erwächst und die Studie minimale Risiken und Belastungen darstellt. Im Falle von SURGE-Ahead trifft

Fall (2) zu. Aktuelle Erkenntnisse aus dem Alterstraumatologischen Zentrum (ATZ) Ulm belegen, dass die Gruppe der kognitiv eingeschränkten PatientInnen besonders vulnerabel für perioperative Komplikationen, Mortalität oder anhaltende funktionelle Defizite ist (13). Sie stellen ca. 40% der betreuten Patient:innen dar. Damit würde dieser Personenkreis in besonderer Weise von einem geriatrischen Co-Management und einer optimalen Nachsorgeentscheidung profitieren.

Sollte sich Patient:innen im Rahmen des Aufklärungsgespräches als nicht-einwilligungsfähig erweisen, erfolgen Aufklärung und Einholung der Einwilligung mit der/dem gesetzlichen Betreuer:in oder der/dem Bevollmächtigten (siehe Anhang: Information für rechtl. Stellvertreter:innen, Einwilligung rechtl. Stellvertreter:in).

Sind bei Nicht-Einwilligungsfähigkeit einer Patientin/eines Patienten keine gesetzlichen Betreuer:innen oder Bevollmächtigten vorhanden, so ist die Studienteilnahme nicht möglich (Ausschlusskriterium). Auch wenn die Vollmacht oder Betreuungsverfügung nicht vorgelegt werden kann, ist die Studienteilnahme nicht möglich. Abbildung 1 zeigt das Vorgehen bei der Rekrutierung von Patient:innen mit Hinweisen auf kognitive Einschränkungen.

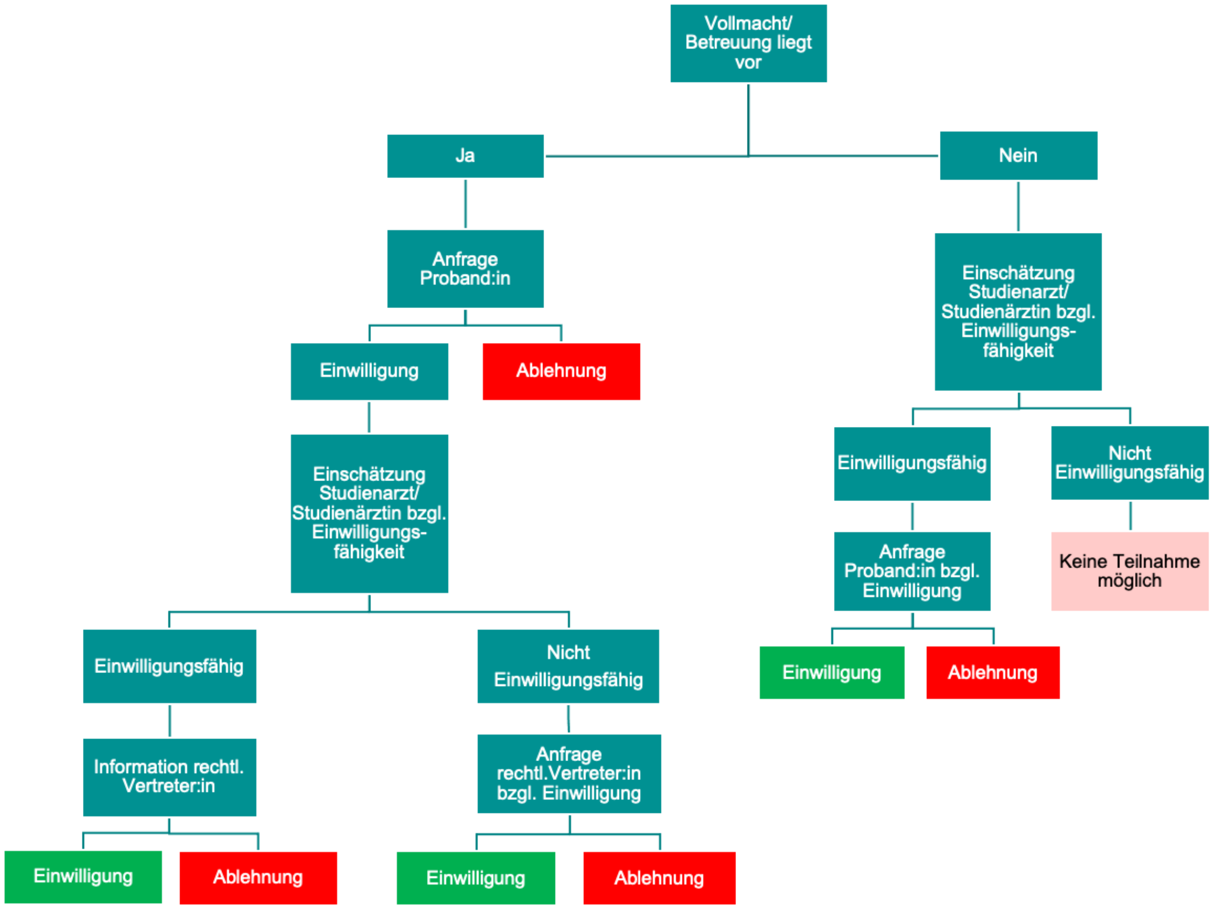

Abbildung 1: Algorithmus Einwilligung bei Patient:innen mit kognitiven Einschränkungen.

6.6 Untersuchungszeitpunkte

Je nach Liegedauer der Proband:innen wird es bis zu sieben Datenerfassungszeitpunkte geben, T0-T6:

- T0: Präoperativ
  - T0.1: Präoperativ zu erhebende Assessments
  - T0.2: Prä- oder postoperativ zu erhebende Assessments (abhängig von der klinischen Situation der Proband:innen)
- T1-T4: Postoperativ stationär Tag 1, 3, 5 und 7 nach OP (bei kürzerer Liegedauer evtl. weniger Assessmentzeitpunkte)
- T5: 1-2 Tage vor Entlassung
- T6: Follow-up 90 Tage nach der Entlassung (± 7 Tage)

Zusätzlich gibt es Erhebungen, die nach der Entlassung durchgeführt werden, hier aber nicht an einen festen Zeitraum gebunden sind (Abbildung 2).

Nach dem Einschluss der Proband:innen werden die präoperativen Assessments (T0) durchgeführt (siehe Kapitel 7). Einige Assessments werden bei allen Proband:innen präoperativ erhoben (T0.1). Diese Assessments können 0-3 Tage vor der Operation durchgeführt werden. Sollte die Operation um mehr als drei Tage verschoben werden, müssen die präoperativen Assessments wiederholt werden. Um der ggf. niedrigeren Belastbarkeit der Proband:innen, insbesondere bei Erhebung des längeren Assessmentabschnitts T0.2, präoperativ Rechnung zu tragen, ist die Möglichkeit vorgesehen, einen Teil der darüber hinaus geplanten präoperativ geplanten Assessments ggf. postoperativ oder durch eine Fremdanamnese zu erheben. An den Tagen 1, 3, 5 und 7 nach der Operation werden die postoperativen Befragungen und Assessments durchgeführt (T1-T4). Hier nimmt die Studienassistenz zunächst jeweils Einsicht in die Patientenakte sowie die Systemdaten (s.u.) und übernimmt relevante Daten. Anschließend erfolgt die Befragung mit den Patient:innen. An Tag 1 nach der Operation wird der Mobilitätssensor (Axivitiy AX6®) angebracht (siehe Kapitel 7.1.5). Sollten Patient:innen vor Tag 7 nach der Operation entlassen werden, werden die postoperativen Assessments nur bis zum Entlasstag durchgeführt. Zur Entlassung werden von der Studienassistenz die Systemdaten erneut erfasst. Zudem erfolgt 0-3 Tage vor der Entlassung die Experteneinschätzung durch die/den Geriatrie-erfahrene/n Ärztin/Arzt basierend auf den erhobenen MGDS Daten, der Krankenakte und einem persönlichen Patientenkontakt inkl. Durchführung der Entlassassessments (T5). Diese Einschätzung wird ausschließlich für das Training der KI genutzt und hat keinen Einfluss auf die tatsächliche Behandlung der Patient:innen. Im Anschluss an die Entlassung erfolgt eine retrospektive Erfassung unerwünschter

Ereignisse und Komplikationen, sowie die kritische Beurteilung des ärztlichen Entlassberichtes hinsichtlich Vollständigkeit und Güte (zeitunabhängig).

Parallel zu den Assessments, die mit den Proband:innen zusammen ausgefüllt werden, werden Daten aus dem Krankenhausinformationssystem (KIS), der Patientenakte und dem Laborinformationssystem (LIS) händisch erfasst. Die Stammdaten aus dem KIS werden hierbei einmalig erfasst. Klinische KIS-Daten (z.B. Medikation, Diagnosen) und die Daten aus dem LIS werden präoperativ und an den Tagen 1, 3, 5 und 7 nach der Operation sowie zur Entlassung erfasst bzw. auf neue Eingaben geprüft und aktualisiert (T1-T5).

90 Tage (± 7 Tage) nach der Entlassung wird ein telefonisches Follow-up (FU) durchgeführt (T6). Dieses nimmt ca. 30-40 Minuten in Anspruch (siehe Kapitel 7). Bei nicht-einwilligungsfähigen Patient:innen wird vorzugsweise der/die Betreuer:in befragt. Sollte diese Person jedoch nicht aus dem Angehörigenkreis sein (Berufsbetreuer:in), wird der/die Proband:in befragt. Zudem wird versucht, die Einschätzung der Hausärzt:innen zu erheben. Abbildung 2 zeigt eine Übersicht der Untersuchungszeitpunkte.

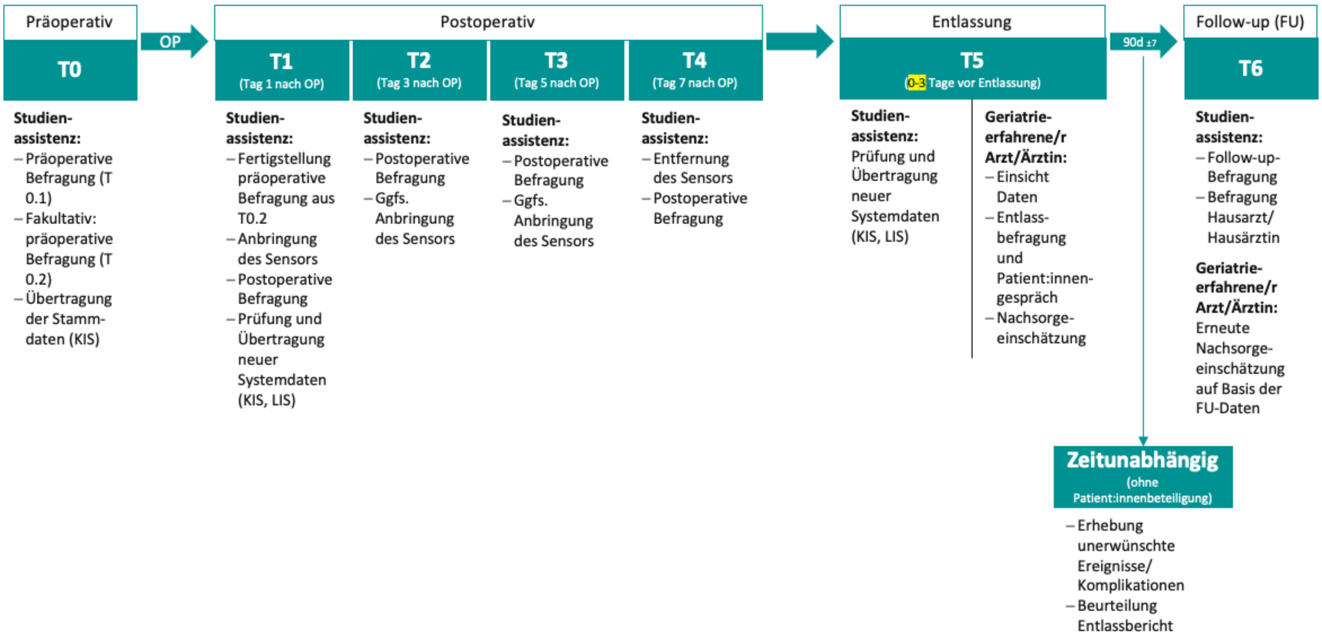

Abbildung 2: Übersicht über die Untersuchungszeitpunkte

7 Datenmanagement

7.1 Datensatz und Fragenkatalog

Für die Entwicklung und Funktionalität des Dashboards wurde ein Datensatz, das MGDS, definiert. Dieser setzt sich zusammen aus Parametern, die für die Behandlung geriatrischer Patient:innen wichtig sind. Die Parameter basieren auf publizierten wissenschaftlichen Erkenntnissen und daraus abgeleiteten klinischen Leitlinien. Die Literatur wurde im Rahmen systematischer Literaturarbeiten gesichtet (14,15). Der Auswahl der Daten und Assessments gingen zudem mehrere Workshops (11/2021 - 05/2022) mit den Kooperationspartnern von SURGE-Ahead und einem internationalen Advisory Board voran.

Das MGDS besteht aus 1) Parametern, die aus existierenden Systemen oder Akten (Krankenhausinformationssystem, Laborinformationssystem, Anästhesiebogen, Patientenakte) extrahiert werden, 2) Daten, die mittels validierter Assessments und Fragebögen erhoben werden und 3) Sensordaten zu Mobilitätsaspekten. Zusätzlich wird im Rahmen der OKIE noch die Einschätzung der/des Geriatrie-erfahrenen Ärztin/Arztes erfasst. Tabelle 2 listet alle Daten, Untersuchungszeitpunkte und die möglichen Quellen (für eine vollständige Übersicht aller Fragen inkl. der Primärquellen siehe Anhang: Erhebungsbogen).

Für die in Tabelle 2 gelisteten Assessments und Fragen mit Patientenkontakt gehen wir insgesamt von einem Zeitaufwand < 2h aus (verteilt auf die einzelnen Erhebungszeitpunkte). Diese Einschätzung basiert zu einem großen Teil auf den Messergebnissen aus dem SURGE-Ahead Prätest (Ethikantrag Nr. 170/22 – Du/Sta, Bescheid vom 30.05.2022) sowie Erfahrungen aus der Literatur und persönlichen Schätzungen. Der ungefähre zu erwartende Aufwand für die Teilnehmenden für die einzelnen Erhebungszeitpunkte ist ebenfalls in Tabelle 2 dargestellt.

Tabelle 2: Übersicht über die zu erhebenden Daten und durchzuführenden Assessments inkl. der Untersuchungszeitpunkte

Domäne	Assessment/ Fragen	Untersuchungszeitpunkt(e)							
		T0.1	T0.2	T1	T2	T3	T4	T5	T6
Assessments mit Patientenkontakt									
Erwarteter zeitl. Aufwand für Teilnehmer:innen (in min)		6	30	12	7	7	8	5	40
Präoperative Assessments mit der/dem Teilnehmer:in									
Screening geriatrische Patienten	ISAR (Identifying Seniors at Risk) *#	x							
Schmerzen	NRS-P (Numeric Rating Scale - Pain) #	x		x	x	x	x		x
Aktivitäten des täglichen Lebens (ADL)	Barthel Index *#	x[1]		x	x	x	x		x

Delir	4AT	x		x	x	x	x		
**Prä- oder postoperative Assessments mit der/dem Teilnehmer:in (je nach klinischer Situation)**									
Soziodemographie	3 Fragen *#		x	(x)					
Sozialanamnese	11 Fragen *#		x	(x)					
Selbstbezogene Gesundheit	5 Fragen*		x	(x)					x
Patienten-zentrierte Ziele	4 Fragen *#		x	(x)					
Kognition	MoCa 5-min (Montreal Cognitive Assessment Test)		x	(x)					x
Depression	PHQ-4 (Patient Health Questionnaire)		x[2]	(x[2])					x[2]
Malnutrition	NRS-N (Nutrition Risk Score Screen) *#		x	(x)					x
Dysphagie	1 Frage *		x	(x)					
Rauchen	1 Frage *#		x	(x)					
Multimedikation	2 Fragen *		x	(x)					x
Sensorik (Sehen/Hören)	3 Fragen *		x	(x)					
Harnkontinenz, Blasenkatheter	2 Fragen *#		x	(x)					x
Stürze	3 Fragen *		x	(x)					
Lebensqualität	EQ 5D 5L, EQ5 VAS		x	(x)					x
Mobilität	New Mobility Score *		x[2]	(x[2])					x
	Charité Mobility Index (CHARMI) #		x[2]	x	x	x	x		x[3]
	Sensordaten			x<br>fortlaufend T1 – T4					
Frailty	CFS (Clinical Frailty Scale)		x[2]	(x[2])				x[2]	
Geriatrische Expert:innen-empfehlung *(erfolgt bei langer Liegedauer ggfs. mehrfach)*	2 Fragen							x	
**Von einigen Teilnehmer:innen auszufüllen (n=30)**									
Bewegungs-tagebuch	Anhang: Bewegungstagebuch			x<br>fortlaufend T1 – T4					
**Telefonischer Fragebogen Follow-up nach 90d**									
Sozialanamnese Follow-Up	9 Fragen *								x
Medizinischer Verlauf Follow-up	8 Fragen *#								x
Gesundheits-ökonomie und Med. Leistungen	CSSRI *								x
Fragen an Hausärztin/ Hausarzt	4 Fragen *								x
Beurteilung Geriater:in Follow-up	3 Fragen								x
**Daten primär aus der Patientenakte**									
Details stationärer Aufenthalt	16 Fragen/ Daten #		x<br>(im Verlauf vervollständigen)						

Vitalparameter	Blutdruck, Puls, Sauerstoffsättigung, Temperatur #			x	x	x	x		
Komorbiditäten 1 *(OP-Daten werden auch für Re-operation(en) erfasst)*	Diagnosen aus Aufnahmebrief Chirurgie und Anästhesiebogen inkl. #<br>- Gewicht/Größe<br>- ASA<br>- OP-Dringlichkeit<br>- Alkoholkonsum<br>- Komorbiditäten		x	(x)					
Komorbiditäten 2	Abfrage von 15 Erkrankungsbildern *#		x	(x)					
Anthropometrie	Gewicht, Größe; BMI *#		x	(x)					x
Wunden/Dekubiti	2 Fragen *#							x	x
Labordaten des stationären Aufenthaltes #	Hämoglobin #							x	
	MCV #							x	
	Leukozyten #							x	
	CRP #							x	
	Kreatinin #							x	
	eGFR n Cockcroft-Gault #							x	
	Natrium #							x	
	Kalium #							x	
	Albumin #							x	
	Multiresistente Keime #							x	
Medikamente #	Nach Patientenkurve							x	x
Allg. Angaben zur Entlassung	Entlassziel 1 Frage #							x	
**Zeitunabhängige Datenerhebung aus Patientenakte**									
Unerwünschte Ereignisse und Komplikationen bei Entlassung	Global Trigger Tool (GTT)							x	
Beurteilung Entlassbriefe	9 Fragen#							x	

[1]Retrospektiv (ca. 2 Wochen vor Aufnahme) und aktueller Zustand
[2]Retrospektiv (ca. 2 Wochen vor Aufnahme)
[3]Zu erheben als Patient Reported Outcome (CHARMI-PROM)
*Ggfs. von Angehörigen oder Bezugspersonen via Fremdanamnese zu erheben (bei vorliegender unterzeichneter Schweigepflichtsentbindung)
#Ggfs. aus der Patientenakte zu erheben

7.1.1 Existierende Datenquellen

Viele Daten, wie beispielsweise Angaben zu Gewicht, Größe, Dekubiti, unerwünschten Ereignissen und Komplikationen, der Medikation (inkl. Frequenz und Dosis), dem Alkohol-/Rauchverhalten und Laborparameter werden bereits routinemäßig erfasst und an verschiedenen Orten/in verschiedenen Systemen dokumentiert. Diese Quellen werden zuerst geprüft, um die Belastung für die Proband:innen so gering wie möglich zu halten. Explizit werden der Anästhesiebogen, die Patientenkurve, der Medikationsplan, die elektronische Patientenakte im Klinikinformationssystem (KIS) und das

Laborinformationssystem (LIS) geprüft. Falls die Daten nicht aus existierenden Quellen extrahierbar sind, werden die Proband:innen gefragt. Die Quelle der Daten wird in allen Fällen dokumentiert.

7.1.2 Assessments und Fragebögen

Der größte Teil des MGDS setzt sich aus klinischen Assessments zusammen, die die wichtigsten Domänen der Behandlung geriatrischer Patient:innen abdecken (z.B. Kognition, Mobilität, Schmerzen, Kontinenz, Aktivitäten des täglichen Lebens oder Komorbiditäten). Darüber hinaus wird die deutsche Version des „Client Sociodemographic and Service Receipt Inventory (CSSRI)“ (16,17) (angepasst an die Projektanforderungen) beim Follow-up T6 genutzt, um die Inanspruchnahme von Gesundheitsdienstleistungen für die gesundheitsökonomische Evaluation zu erfassen. Einige Assessments und Fragen werden mehrfach erhoben, um eine Verlaufsdokumentation zu ermöglichen. Wenn erforderlich können einzelnen Assessments und Fragen auch via Fremdanamnese von Angehörigen erhoben werden (siehe Tabelle 2).

7.1.3 Geriatrische Experteneinschätzung

Nachsorgeentscheidung: Die Expertenempfehlung durch eine/n Geriatrie-erfahrene Ärzt:in erfolgt bei T5 (Entlassung) und bei T6 (Follow-up). In diesem Zusammenhang wird basierend auf den zur Entlassung aus der Akutklinik erhobenen Assessmentdaten des MGDS, der Krankenakte und einem persönlichen Gespräch mit den Proband:innen eine Empfehlung zur bestmöglichen Nachsorgeoption dokumentiert (hierbei erfolgt keine Interaktion mit dem klinischen Behandlungsteam). Diese wird dann anhand der retrospektiven Betrachtung des Weiteren Verlaufs beim Follow-up-Termin erneut bestätigt oder angepasst. Bei langer Liegedauer oder akuter Verschlechterung nach T5, kann die Experteneinschätzung ggfs. mehrfach erfolgen.

7.1.4 Zeitunabhängiges Review der Patientenakte

Die Bedürfnisse geriatrischer Patient:innen sind oft komplex und fachübergreifend. Um den stationären Behandlungsverlauf der teilnehmenden Proband:innen hinsichtlich des Bedarfs an geriatrischem Co-Management zu analysieren sollen verschiedene Inhalte der Patientenakte kritisch analysiert werden. Diese Analyse erfolgt retrospektiv und zeitunabhängig von der stationären Behandlung der Proband:innen.

1. Bewertung der medizinischen Entlassberichte: Der medizinische Entlassbericht (Arztbrief) ist das wichtigste Dokumentationsinstrument, um die Quintessenz des stationären Behandlungsverlauf darzustellen und eine nahtlose Fortführung der medizinischen Betreuung in einem weiterführenden Versorgungssetting zu gewährleisten. Es stellt ein wesentliches Kommunikationsmittel zwischen verschiedenen Sektoren des Gesundheitssystems dar und ist ein wichtiger Aspekt einer gelungenen Continuity of Care (COC). Um die inhaltliche Güte der

Entlassberichte darzustellen wird im Verlauf eine standardisierte Bewertung mit einem Scoring System durchgeführt (18).

2. Unerwünschte Ereignisse im Behandlungsverlauf: Aufgrund ihrer Multimorbidität und Gebrechlichkeit sind geriatrische Patient:innen für unerwünschte Ereignisse (Adverse Events, AE) besonders anfällig. Zur Darstellung der Rate chirurgischer und nicht-chirurgischer AEs und Komplikationen werden diese mittels einer retrospektiven Akteneinsicht basierend auf dem Global Trigger Tool (GTT) erhoben (19).

7.1.5 Aktivitätsmessung mit dem Axivity AX6®-Sensor

Mobilität und körperliche Aktivität sind wesentliche Voraussetzungen für Lebensqualität, selbstständige Versorgung und unabhängige Lebensführung im Alter. Immobilität stellt dagegen einen wichtigen Behinderungsgrund dar, der oftmals zu Pflegebedürftigkeit und Abhängigkeit im Alter führt. Um die körperliche Aktivität und Mobilität im Zusammenhang mit Operationen besser zu verstehen, soll zusätzlich zu den Assessments eine Aktivitätsmessung mittels eines am unteren Rücken (lumbal) angebrachten sechsaxialen Beschleunigungssensors (AX6®, Fa. Axivity Ltd., Newcastle, United Kingdom) durchgeführt werden (siehe Anhang: Datenblatt AX6). Der Sensor hat eine amtliche Zulassung und ein CE-Zertifikat und wurde am Institut für Geriatrische Forschung bereits in anderen Projekten mit geriatrischen Patient:innen ohne Nebenwirkungen eingesetzt (z.B.: von der Ethikkommission der Universität Ulm genehmigte Prometheus-Studie (Ethikvotum Nr. 26/21 / Jahr 2021)).

Der Sensor wird an Tag 1 (T1) nach der Operation mit zwei wasserdichten Folien (OPSITE FLEXIFI PNZ: 07478029) auf Höhe der Taille (Lendenwirbel 5) mittig am Rücken angebracht (siehe Abb. 3). Die Höhe der Anbringung wird dokumentiert (Ferse bis Oberkante Sensor; Messung im Liegen). Die Körperhygiene (Waschen, Duschen) ist weiterhin uneingeschränkt möglich. Sollte die Anbringung aufgrund der körperlichen Verfassung zu T1 noch nicht möglich sein, kann der Sensor ab T2 auch am Oberschenkel angebracht werden. Die Anbringung am Oberschenkel ist auch möglich, wenn die Teilnehmenden den Sensor an der Wirbelsäule ablehnen. Der Sensor wird je nach stationärer Aufenthaltsdauer und Tag der Anbringung bis zu 7 Tage getragen und spätestens zu T4 entfernt. Die Messung wird immer zu Beginn eines vollen Tages gestartet und zum Ende eines vollen Tages beendet. Sie umfasst somit bis zu fünf vollständige Tage. An den unvollständigen Tagen der Anbringung und Entfernung wird keine Messung durchgeführt. Daten, die durch den AX6 erfasst werden, sind beispielsweise Schrittanzahl pro Tag, Dauer der Liege-, Steh-, und Gehzeiten in min pro Tag und die Anzahl der Bewegungsabläufe Liegen-Sitzen und Sitzen-Stehen.

Zur Auswertung wird unter anderem das frei verfügbare GGIR-Paket (Raw Accelerometer Data Analysis) für R genutzt (20). Um diese und möglicherweise weitere Algorithmen für das

Patientenkollektiv der OKIE zu prüfen, füllen 30 Teilnehmende (N=10 pro rekrutierender Klinik) während des Tragens des Sensors (T1 – T4) ein Bewegungstagebuch aus. Kognitiv eingeschränkte Teilnehmer:innen sind hiervon ausgeschlossen. Hier werden u.a. die Zeiten des Aufstehens am Morgen und Zubettgehens am Abend erfasst (Anhang: Information Bewegungstagebuch, Bewegungstagebuch).

7.2 Datenerfassung

Die Datenerfassung wird von Studienassistent:innen durchgeführt. Je Klinik ist hierfür eine Stelle vorgesehen (die sich auch gegenseitig unterstützen sollen). Alle drei Kliniken bzw. die Studienassistent:innen werden für die Datenerhebung mit einem 2in1-Gerät (Laptop und Tablet) der Firma Dell ausgestattet. Das Gerät wird im sicheren Kliniknetz betrieben und verfügt über einen Zugang zum KIS und LIS. Es wird in Kooperation mit dem Zentrum für Information und Kommunikation des Universitätsklinikums Ulm betrieben und erfüllt die strengen IT-Voraussetzungen für den Betrieb im Netz des Universitätsklinikums Ulm.

Für die OKIE wurde eine eigene Anwendung für die Datenerfassung programmiert, die innerhalb des Netzwerkes des Universitätsklinikums Ulm gehostet wird und auf die über jeden Standardbrowser (z.B. Edge, Chrome, Firefox) innerhalb des Netzwerks zugegriffen werden kann. Zur Dateneingabe rufen die Studienassisten:innen über eine sichere Verbindung die Anwendung in ihrem Browser auf und wählen sich über ihre individuell zugewiesenen Zugangsdaten in die Oberfläche ein. Über voreingestellte und an die standardisierten Fragebögen angepasste Eingabemasken werden die Daten von den Studienassistent:innen erfasst (siehe Abb. 4 und Anhang: Eingabemaske). Die Erfassung der Daten erfolgt mittels Maus und Tastatur oder über den Touchscreen des Gerätes. Daten, die ohne eine Beteiligung der Proband:innen oder deren Angehörigen erfasst werden können (d.h. Daten aus dem KIS oder LIS), werden von den Studienassistent:innen an einem Arbeitsplatz im Universitätsklinikum Ulm extrahiert und in der Eingabemaske eingetragen. Vor jedem Assessment werden die Bedingungen während der Befragung sowie der Isolationsstatus des/der Patient:in erfasst (siehe Anhang: Erhebungsbogen). Alle über die Eingabemaske erfassten Daten werden in einer passwortgeschützten Datenbank gespeichert, die auf demselben Server liegt, wie die Anwendung.

Mittels regelmäßiger Projektmonitorings erfolgt eine laufende Überwachung des Projektfortschritts. Hierbei wird auch die Datenqualität geprüft, um Probleme rechtzeitig zu identifizieren und gegensteuern zu können.

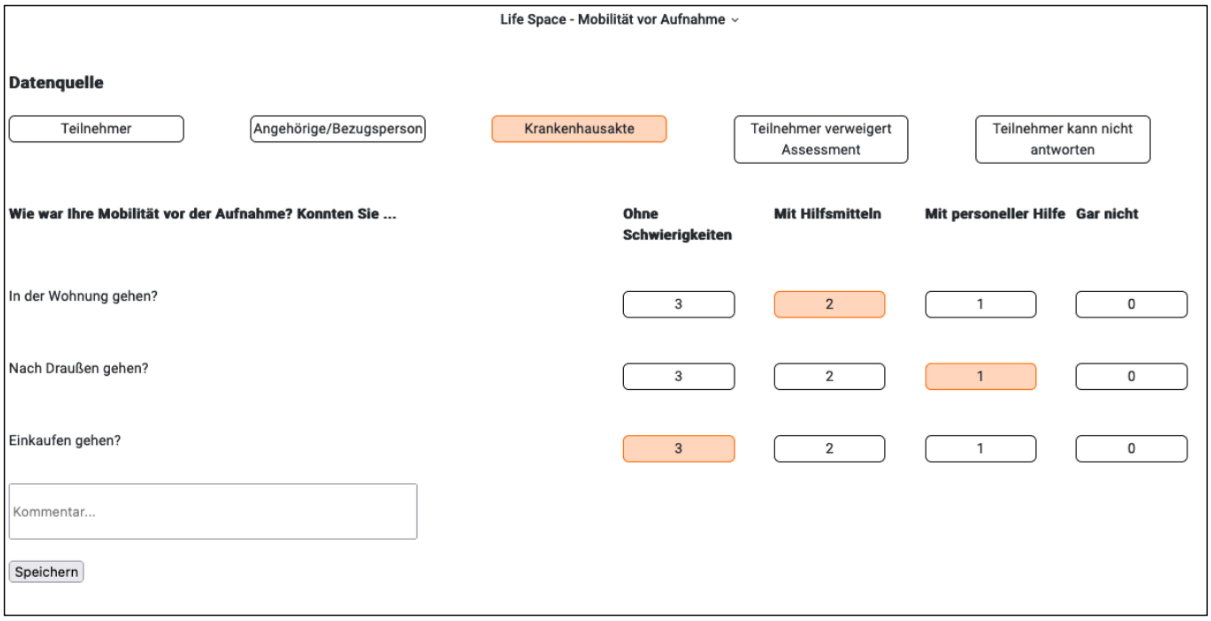

Abbildung 4: Beispiel für die Ansicht der Studienassstent:innen bei der Erfassung der Daten (hier: New Mobility Score) (für weitere Ansichten siehe Anhang: SURGE-Ahead Eingabemaske)

7.3 Datenauswertung

Primär ist das Ziel der aktuellen OKIE die Erhebung eines Trainingsdatensatzes für die KI. In den Jahren 4-6 des SURGE-Ahead-Projektes ist eine Interventionsstudie zur Erprobung des Dashboards im klinischen Betrieb geplant. Die OKIE dient daher auch zur Vorbereitung sowie als Vergleichskohorte für die Evaluation der Intervention. Im Folgenden werden die wichtigsten Outcome-Parameter aufgeführt.

7.3.1 Programmierung der KI für die Nachsorgeempfehlung

Ziel des gesamten SURGE-Ahead-Projektes ist es, eine digitale Anwendung zu entwickeln, die das perioperative geriatrische Co-Management abbildet und eine Empfehlung für geeignete Nachsorgeeinrichtungen ausspricht. Für diesen zweiten Aspekt gehen wir derzeit im deutschen Gesundheitssystem von folgenden Hauptkategorien (Klassen) aus:

- Geriatrische Akutklinik
- Rehaeinrichtung
  - Fachspezifische Rehaeinrichtung
  - Stationäre geriatrische Rehaeinrichtung (hauptsächlich)
  - Ambulante geriatrische Rehaeinrichtung (kaum)
- Pflegeheim
- Nach Hause
  - ohne personeller Hilfe
  - mit personeller Hilfe

Darüber hinaus kann es sein, dass einige wenige Patient:innen in eine andere Akutklinik, eine alterspsychiatrische Einrichtung, oder in ein Hospiz verlegt werden. Da die größte Bandbreite dieser Nachsorgeoptionen in der Klinik für Unfall-, Hand-, Plastische und Wiederherstellungschirurgie genutzt wird, werden diese Patient:innen bei der Rekrutierung übergewichtet. In der Allgemein- und Viszeralchirurgie sowie der Urologie werden nach Schätzungen der Projektpartner ca. 70-90% der Patient:innen nach Hause entlassen.

Um die Nachsorgeeinrichtung möglichst genau vorhersagen zu können, sind für jede Nachsorgeeinrichtung (Klasse) ausreichend Datenpunkte notwendig. Die genaue Anzahl der benötigten Datenpunkte insgesamt und pro Klasse variiert mit dem verwendeten Model (z.B. linear und nicht-linear) und der angestrebten Performanz. Da das Model post-hoc und explorativ bestimmt wird, ist eine möglichst gleichmäßige Verteilung der Datenpunkte auf die einzelnen Klassen anzustreben. Es wird ein Datensatz benötigt, in dem die zu vorhersagenden Kategorien oft genug auftreten (11). Das Ziel für die OKIE ist daher zunächst ein Ansatz mit vier Kategorien, und zwar Entlassung 1) in eine geriatrische Akutklinik, 2) in eine Rehaklinik, 3) in ein Pflegeheim und 4) nach Hause. Wenn möglich, werden mit dem erhobenen Datensatz anschließend weitere Berechnungen zur Verfeinerung durchgeführt.

7.3.2 Deskription des aktuellen Behandlungsstandards

Der OKIE-Datensatz erfasst den aktuellen Behandlungsstandard und soll den Bedarf für ein geriatrisches Co-Management hinsichtlich verschiedener primärer und sekundärer Endpunkte bewerten. Aktuell sind insb. Methoden der deskriptiven Statistik zur Beschreibung der verschiedenen Endpunkte geplant. Hierzu gehört auch die gesundheitsökonomische Betrachtung. Das Kosten-Effektivitätsverhältnis der eingesetzten Ressourcen wird aus volkswirtschaftlicher Perspektive basierend auf den der Inanspruchnahme von Gesundheitsleistungen (CSSRI) und der Lebensqualität (EQ 5D 5L) nach der Nettonutzenmethode bestimmt. Die Schätzung der Krankheitskosten erfolgt durch die Multiplikation der in Anspruch genommenen Leistungseinheiten mit den ermittelten Kosten dieser Einheiten, jeweils für einen Zeitraum von 3 Monaten (T0 bis T6). Für die in einem zukünftigen Projektabschnitt geplante Interventionsstudie sollen die erhobenen Daten ggf. als Vergleichskohorte genutzt werden. Für diese Interventionsstudie wird im Verlauf ein separater Ethikantrag gestellt werden.

7.3.3 Vervollständigung des Datensatzes

Anhand der erhobenen Daten werden übergreifende Scores berechnet und mit den Daten verifiziert, um diese dann ggf. auch in der geplanten Interventionsstudie einzusetzen. Dazu gehört beispielsweise der Nottingham Hip Fracture Score zur Vorhersage der 30-Tage Mortalität nach einer Hüftfraktur. Der Score basiert auf den folgenden sieben Parametern: Alter, Geschlecht, Anzahl der Komorbiditäten,

kognitiver Status, präoperative Wohnsituation, Hämoglobinkonzentration bei Aufnahme und dem Vorhandensein von bösartigen Tumoren (21). Weiterhin wird mit den Daten der Delir-Risikoscore und Frailty-Index aus der PAWEL-Studie (22) (das Forschungsteam des Instituts für Geriatrische Forschung war an dieser Studie ebenfalls beteiligt) in einer für das MGDS angepassten Form validiert.

Der medikamentöse Behandlungsplan wird hinsichtlich einer Über- aber auch Untertherapie sowie potentiell inadäquater Medikamente (PIM) für Ältere überprüft. Hierzu wird der medikamentöse Behandlungsplan zu Beginn der stationären Behandlung, zu ihrem Abschluss sowie zum Follow-up Zeitpunkt mit Hilfe validierter Tools (u.a. FORTA) überprüft (23). Die Optimedis AG unterstützt die automatisierte Berechnung des FORTA-Scores. Die Auswertung erfolgt über die Optimedis-Software, die in einem Docker zur Verfügung gestellt wird. Ausschließlich die Studienidentifikationsnummer, das Alter, die ICD- und ATZ-Codes werden für die Analyse benötigt.

7.3.4 Methodische Anpassungen

Im Rahmen der OKIE soll geprüft werden, ob alle gewählten Assessmentzeitpunkte (v.a. für die Verlaufsassessments) auch in der späteren Interventionsstudie nötig sind, oder ob hier noch Anpassungen vorgenommen werden sollten (z.B. Streichung des Assessmentzeitpunktes T2 aufgrund fehlender oder nur geringer Änderung). Auch weitere methodische Anpassungen sind denkbar.

8 Unerwünschte Ereignisse (AE) / schwerwiegende unerwünschte Ereignisse (SAE)

8.1 (S)AE für Teilnehmende

Sollten im Rahmen der Befragung AE oder SAE auftreten, wird die Studienzentrale über den Vorfall informiert. Anschließend wird geprüft, ob ein Zusammenhang mit der Studie besteht. Ist dies der Fall, wird die zuständige Ethikkommission innerhalb von 7 Arbeitstagen informiert. Durch das klinische Behandlungsteam vor Ort kann jederzeit eine optimale Therapie und Versorgung gewährleistet werden. Darüber hinaus kann bei unerwünschten Ereignissen zu jedem Zeitpunkt auf die geriatrische Expertise aus dem Studienteam zurückgegriffen werden.

8.2 Unerwünschte Ereignisse, die den Studienerfolg gefährden

Um für mögliche Eventualitäten und unerwünschte Ereignisse, die den Studienfortschritt und -erfolg gefährden können, vorbereitet zu sein, wurden verschiedene Maßnahmen definiert. Diese sind in Tabelle 3 festgehalten.

Tabelle 3: Risiken für den Studienerfolg und Maßnahmen

**Risiken**	**Maßnahmen**
Rekrutierungsrate zu gering	Präventiv:<br>- Informationsveranstaltung für klinisches Personal<br>- Umfassende Einweisung der Studienassistent:innen<br>Reaktiv:<br>- Verlängerung des Rekrutierungszeitraumes von 9 auf 12 Monate<br>- Rekrutierung von Patient:innen mit einem ISAR ≥ 1
Datenverlust	Präventiv:<br>Regelmäßiges lokales und dezentrales Backup der gesamten Datenbank<br>Reaktiv:<br>Wiederherstellung der Datenbank aus dem Backup
Ausfall von Untersuchungszeitpunkten (z.B. wg. Urlaub, Erkrankung, nicht-Verfügbarkeit der Proband:innen)	- Vertretung durch andere Studienassistent:innen oder im Notfall durch die Studienleitung.<br>- Wenn keine Vertretung/keine Befragung möglich:<br>o Einzelne Verlaufsassessments (T1-T4) können im Notfall ausfallen<br>o T0, T5 und T6 müssen nach Möglichkeit nachgeholt werden (dann mit entsprechendem Vermerk)
Abläufe uneinheitlich zw. Kliniken	- Zu Beginn regelmäßige Telefonkonferenz zwischen dem Projektteam, den Studienärzt:innen und den Studienassisstent:innen

	- Regelmäßige Prozessmonitoring durch die Studienleitung<br>- Regelmäßiges Datenmonitoring durch Mitglieder der Studienleitung
Drop-outs höher als erwartet	Erhöhung der Rekrutierungszahl
Gefahr des Verzugs der Gesamtstudie	Review der Patientenakte und Entlassberichte mit einer reduzierten Anzahl von Studienteilnehmer:innen durchführen oder komplett streichen
Covid-19	Erhöhte Hygiene- und Vorsichtsmaßnahmen (siehe Anhang: Hygienekonzept)

9 Ethische und rechtliche Aspekte

9.1 Einwilligung

Alle Teilnehmenden werden mündlich und schriftlich über die Studienziele, den Ablauf, mögliche Risiken und die Nutzung der Daten informiert (siehe Anhang: Information für Patientinnen und Patienten). Die Teilnehmenden werden explizit darauf hingewiesen, dass sie bei Problemen oder Fragen das Studienteam kontaktieren oder auch ihre Teilnahme an der Studie jederzeit beenden können. Die Kontaktinformationen des Studienpersonals (Telefonnummer, E-Mail-Adresse) erhalten die Teilnehmenden mit den Aufklärungsunterlagen.

Es werden Einwilligungen eingeholt für die Studienteilnahme, die Fremdanamnese durch Angehörige/Bezugspersonen und Hausärzt:innen (Schweigepflichtentbindung) sowie die Erfassung der Mobilitätsdaten durch den Sensor. Die Teilnehmenden bestätigen ihre Einwilligung mit ihrer Unterschrift während des Aufklärungsgesprächs.

Für nicht-einwilligungsfähige Patient:innen wird ein/eine gesetzliche Betreuer/in hinzugezogen (siehe auch Kapitel 6.4).

9.2 Kosten und Entschädigung für Teilnehmende

Für die Teilnehmenden entstehen keine Kosten. Die Teilnehmenden erhalten keine monetäre oder anderweitige Entschädigung.

9.3 Risiken für Teilnehmende

Die Risiken für die Teilnehmenden sind gering. Sollte die Befragung für die Patient:innen eine zu große Belastung darstellen, wird diese umgehend unterbrochen und ggf. zu einem späteren Zeitpunkt fortgesetzt. Da dies nach unserem Ermessen eher bei den präoperativen Assessments passieren könnte (v.a. im Falle von Notfallpatient:innen), werden hier nur die vier wichtigsten Domänen abgefragt (Identifikation geriatrischer Patientinnen und Patienten (ISAR), Delir (4AT), ADL (Barthel), Schmerzen (NRS-P)). Der ISAR-Score wird am Universitätsklinikum Ulm bereits in den meisten Fällen routinemäßig erhoben. Die Schmerzskala und der Barthelindex werden in allen Kliniken genutzt. Da der ISAR-Score Teil des Screenings ist, kommt i.d.R. nur der 4AT-Test präoperativ dazu. Hierfür ist ein zusätzlicher Zeitaufwand von etwa zwei Minuten zu erwarten.

Das Risiko, dass durch die Teilnahme psychische Beschwerden hervorgerufen werden, wird als gering eingeschätzt. Ggf. kann dann der längere präoperative Assessmentabschnitt (T0) in 2 Abschnitte (T0.1 und T0.2) unterteilt werden, natürlich ist ein Rücktritt von der freiwilligen Studienteilnahme durch die Proband:innen jederzeit möglich.

Das Risiko einer Sars-Cov2-Infektion durch den Kontakt zum Studienpersonal wird ebenfalls als gering eingestuft. Das gesamte Studienpersonal unterliegt der Pflicht, sich regelmäßig mittels Antigenschnelltests zu testen sowie für die Dauer des Aufenthaltes im Universitätsklinikum Ulm eine FFP2-Maske zu tragen. Dem Studienpersonal wird zudem ausreichend Desinfektionsmittel zur Verfügung gestellt (siehe Anhang: Hygienekonzept). Darüber hinaus verfügt das Universitätsklinikum Ulm über ein eigenes, umfangreiches Hygienekonzept, nachdem sich das Studienpersonal zu richten hat. Auch ggf. im weiteren Verlauf stattfindende Anpassungen des Hygienekonzepts des Universitätsklinikums Ulm (z.B. höherfrequente Antigenschnelltestungen für Mitarbeitende) werden durch das Studienpersonal umgesetzt.

Alle Teilnehmenden können jederzeit und ohne Angabe von Gründen von der Studie zurücktreten.

9.4 Nutzen

Auf Wunsch können die gewonnenen Erkenntnisse nach Abschluss der Studie an die Proband:innen weitergegeben werden. Darüber hinaus leisten die Proband:innen einen wertvollen Beitrag zum wissenschaftlichen Erkenntnisgewinn.

Während der stationären Studienphase stehen die Studienärzt:innen und -assistent:innen für Fragen zur Verfügung.

9.5 Versicherung

Während der Teilnahme an der Studie besteht für alle Proband:innen Versicherungsschutz.

Das Universitätsklinikum Ulm und das an der Studie mitwirkende Personal sind haftpflichtversichert für den Fall, dass die Proband:innen durch deren Verschulden einen Schaden erleiden.

9.6 Datenschutzkonzept

9.6.1 Pseudonymisierung der Studienteilnehmenden

Nach Prüfung der Einschlusskriterien, Aufklärung und Einwilligung der Proband:innen legt die/der Studienassistent:in einen neuen Eintrag in der Studiendatenbank an. Dadurch wird automatisch eine Studienidentifikationsnummer generiert. Diese setzt sich zusammen aus einer Identifikationsnummer der beteiligten Fachabteilung (UCH 11, AVC 12, URO 13) und einer fortlaufenden Nummer der eingeschlossenen Patienten. Hieraus ergibt sich folgendes Pseudonymisierungsformat:

- für UCH: 11001, 11002, ...
- für AVC: 12001, 12002, ...
- für URO: 13001, 13002, ...

In der Studiendatenbank werden keine personenbezogenen Daten wie Name, Adresse oder Telefonnummer festgehalten. Zur Entschlüsselung der pseudonymisierten Datensätze wird eine Studienteilnehmenden-Identifikationsliste mit allen teilnehmenden Patient:innen handschriftlich geführt (siehe Anhang: Studienteilnehmenden-Identifikationsliste). Die Studienteilnehmenden-Identifikationsliste beinhaltet die Studienidentifikationsnummer, den vollständigen Namen, das Geburtsdatum, die Adresse und die Telefonnummer. Zusätzlich werden ggf. die Kontaktdaten einer/eines Angehörigen oder sonstigen Bezugsperson oder, bei nicht-einwilligungsfähigen Personen, der bevollmächtigten Person, sowie der/des Hausärztin/Hausarztes hinterlegt. Für alle Proband:innen wird das Einschlussdatum und ggf. Abbruchdatum dokumentiert. Die papierbasierte Identifikationsliste ist die einzige Möglichkeit, die Datensätze in der Studiendatenbank einer Person zuzuordnen. Die Daten in der Liste werden genutzt, um teilnehmende Patient:innen zu identifizieren, Unklarheiten zu klären oder um die erfassten Daten den Proband:innen auf deren Wunsch hin auszuhändigen. Die Identifikationslisten verbleiben in den jeweiligen Kliniken in verschlossenen Schränken in zugangsbeschränkten Räumen. Die Identifikationslisten werden vom autorisierten Studienpersonal vor Ort aktualisiert und gepflegt.

9.6.2 Datenerfassung über die Eingabemaske

Die Datenerfassung erfolgt über eine speziell programmierte Benutzeroberfläche. Wie unter 7.2 aufgeführt, erfolgt die Kommunikation zwischen Studienassisten:innen und Anwendung verschlüsselt und passwortgeschützt. Die Eingebenden Daten werden in einer passwortgeschützten Datenbank abgelegt, von der ein regelmäßiges Backup erstellt wird. Die Daten verbleiben bis zum Studienabschluss nur innerhalb des Netzwerks des Universitätsklinikums.

9.6.3 Datenerfassung durch die AX6®-Sensoren

Alle erhobenen Daten werden lokal auf der Speicherkarte des Sensors gespeichert. Nach Ablauf der Tragezeit wird der Sensor entfernt und die Daten durch die Studienassistenz ausgelesen. Die Sensordaten werden zunächst, versehen mit der eindeutigen Studienidentifikationsnummer, in einer eigenen Datenbank gespeichert. Erst nach der Auswertung der Rohdaten werden diese in die Studiendatenbank eingespeist. Der Sensor wird hygienisch gereinigt, geladen, neu kalibriert und kann nachfolgend erneut in der Studie genutzt werden.

9.6.4 Aufbewahrung und Archivierung der Daten

Die studienbezogenen, pseudonymisierten Daten verbleiben bis zum Studienabschluss auf dem Server des Universitätsklinikums Ulm. Anschließend werden die Daten auf einem gesicherten Datenträger (z.B. USB-Stick) in die Studienzentrale an der AGAPLESION Bethesda Klinik Ulm überführt, wo sie auf den Server des Instituts für Geriatrische Forschung übertragen und in einer durch das Institut-betriebenen Cloud abgelegt werden. Dieser ist durch eine Web Application Firewall (WAF) vor

unbefugtem Zugriff geschützt. Der Zugriff ist nur durch eine 2-Faktor-Authentifizierung durch autorisierte Mitarbeitende des Instituts möglich. Die Daten werden entsprechend geltendem Recht mindestens 10 Jahre lang gespeichert. Die Einwilligungserklärungen verbleiben in den jeweiligen Kliniken und werden getrennt von den Studiendaten ebenfalls für 10 Jahre in einem verschlossenen Schrank, in einem verschlossenen, zur Datenaufbewahrung geeigneten Raum aufbewahrt. Die Studienteilnehmenden-Identifikationsliste mit den Pseudonymisierungscodes der Studienteilnehmer:innen verbleibt ebenfalls in der jeweiligen Klinik und werden gemeinsam mit den Einwilligungserklärungen sicher verschlossen verwahrt. Die Identifikationslisten werden nach Abschluss der Studie und vollständiger Datenbereinigung vernichtet. Die Datenbereinigung wird vom autorisierten Studienpersonal der Universität und des Universitätsklinikums Ulm durchgeführt.

9.6.5 Datenschutz und Schweigepflicht

Alle am Projekt beteiligten Mitarbeitenden werden vorab über den verantwortungsvollen Umgang mit den erhobenen Studiendaten und zur Einhaltung der Datenschutzrichtlinien geschult und verpflichten sich hierzu schriftlich mit Projekteintritt (siehe Anhang: Verschwiegenheitserklärung). Alle ärztlichen Projektmitarbeitenden unterliegen der medizinischen Schweigepflicht.

Der Zugriff auf das Programm zur Datenerhebung ist nur mit persönlicher Kennung und Passwort durch die an der Datenerhebung der Studie beteiligten Mitarbeitenden sowie der Studienleitung möglich.

Der Zugang zum Server des Instituts für Geriatrische Forschung ist durch eine 2-Faktor-Authentifizierung geschützt. Nur autorisiertes Studienpersonal mit persönlicher Kennung und Passwort hat Zugang zum Studienserver. Durch die zugewiesene Rolle kann das Studienpersonal nur die Projekte bzw. Daten bearbeiten und einsehen, für die es eine Berechtigung besitzt.

Literatur

1. Rapp K, Becker C, Todd C, Rothenbacher D, Schulz C, König HH, u. a. The Association Between Orthogeriatric Co-Management and Mortality Following Hip Fracture. Dtsch Arzteblatt Int. 24. Januar 2020;117(4):53–9.

2. Grigoryan KV, Javedan H, Rudolph JL. Ortho-Geriatric Care Models and Outcomes in Hip Fracture Patients: A Systematic Review and Meta-Analysis. J Orthop Trauma. März 2014;28(3):e49–55.

3. Shahrokni A, Tin AL, Sarraf S, Alexander K, Sun S, Kim SJ, u. a. Association of Geriatric Comanagement and 90-Day Postoperative Mortality Among Patients Aged 75 Years and Older With Cancer. JAMA Netw Open. 19. August 2020;3(8):e209265.

4. Falaschi P, Marsh D, Herausgeber. Orthogeriatrics: The Management of Older Patients with Fragility Fractures [Internet]. Cham: Springer International Publishing; 2021 [zitiert 8. Februar 2022]. (Practical Issues in Geriatrics). Verfügbar unter: http://link.springer.com/10.1007/978-3-030-48126-1

5. Kmietowicz Z. Emergency laparotomy: lack of geriatrician input leaves frail patients at double risk of death. BMJ. 13. November 2020;371:m4437.

6. Braude P, Goodman A, Elias T, Babic-Illman G, Challacombe B, Harari D, u. a. Evaluation and establishment of a ward-based geriatric liaison service for older urological surgical patients: Proactive care of Older People undergoing Surgery (POPS)-Urology. BJU Int. Juli 2017;120(1):123–9.

7. Ellis G, Gardner M, Tsiachristas A, Langhorne P, Burke O, Harwood RH, u. a. Comprehensive geriatric assessment for older adults admitted to hospital. Cochrane Database Syst Rev. 12. September 2017;9:CD006211.

8. Eamer G, Taheri A, Chen SS, Daviduck Q, Chambers T, Shi X, u. a. Comprehensive geriatric assessment for older people admitted to a surgical service. Cochrane Database Syst Rev. 31. Januar 2018;1:CD012485.

9. Warburton RN, Parke B, Church W, McCusker J. Identification of seniors at risk: process evaluation of a screening and referral program for patients aged ≥75 in a community hospital emergency department. Int J Health Care Qual Assur. 1. Januar 2004;17(6):339–48.

10. McCusker J, Bellavance F, Cardin S, Trépanier S, Verdon J, Ardman O. Detection of older people at increased risk of adverse health outcomes after an emergency visit: the ISAR screening tool. J Am Geriatr Soc. Oktober 1999;47(10):1229–37.

11. Cover TM. Geometrical and Statistical Properties of Systems of Linear Inequalities with Applications in Pattern Recognition. IEEE Trans Electron Comput. 1965;3(EC-14):326–34.

12. World Medical Association. World Medical Association Declaration of Helsinki: Ethical Principles for Medical Research Involving Human Subjects. JAMA. 27. November 2013;310(20):2191–4.

13. Schuetze K, Eickhoff A, Rutetzki KS, Richter PH, Gebhard F, Ehrnthaller C. Geriatric patients with dementia show increased mortality and lack of functional recovery after hip fracture treated with hemiprosthesis. Eur J Trauma Emerg Surg Off Publ Eur Trauma Soc. Juni 2022;48(3):1827–33.

14. Kocar Thomas, Denkinger M, Dallmeier D, Fotteler M, Leinert C. Evidence-based recommendations for acute orthogeriatric care: a systematic review of clinical practice guidelines. PROSPERO 2022 CRD42022292141 [Internet]. [zitiert 12. August 2022]; Verfügbar unter: https://www.crd.york.ac.uk/prospero/display_record.php?ID=CRD42022292141

15. Leinert C, Fotteler M, Kocar T, Dhayana.Dallmeier, Denkinger M. Predictors and outcomes of interest of continuity of care decisions for older inpatients in acute care settings: a scoping review. 24. März 2022 [zitiert 12. August 2022]; Verfügbar unter: https://osf.io/yjzax

16. Chisholm D, Knapp MR, Knudsen HC, Amaddeo F, Gaite L, van Wijngaarden B. Client Socio-Demographic and Service Receipt Inventory--European Version: development of an instrument for international research. EPSILON Study 5. European Psychiatric Services: Inputs Linked to Outcome Domains and Needs. Br J Psychiatry Suppl. 2000;(39):s28-33.

17. Roick C, Kilian R, Matschinger H, Bernert S, Mory C, Angermeyer MC. Die deutsche Version des Client Sociodemographic and Service Receipt Inventory. Psychiatr Prax. Oktober 2001;28(Sup. 2):84–90.

18. Savvopoulos S, Sampalli T, Harding R, Blackmore G, Janes S, Kumanan K, u. a. Development of a quality scoring tool to assess quality of discharge summaries. J Fam Med Prim Care. April 2018;7(2):394–400.

19. IHI Global Trigger Tool for Measuring Adverse Events (Second Edition) | IHI - Institute for Healthcare Improvement [Internet]. [zitiert 1. August 2022]. Verfügbar unter: https://www.ihi.org/resources/Pages/IHIWhitePapers/IHIGlobalTriggerToolWhitePaper.aspx?PostAuthRed=/resources/_layouts/download.aspx?SourceURL=/resources/Knowledge%20Center%20Assets/IHIWhitePapers%20-%20IHIGlobalTriggerToolforMeasuringAdverseEventsSecondEdition_ab736cac-935b-46e4-b6fe-7f98c45f8a78/IHIGlobalTriggerToolWhitePaper2009.pdf

20. Hees VT van, Migueles JH, Sabia S, Patterson MR, Fang Z, Heywood J, u. a. GGIR: Raw Accelerometer Data Analysis [Internet]. 2022 [zitiert 11. Januar 2023]. Verfügbar unter: https://CRAN.R-project.org/package=GGIR

21. Olsen F, Lundborg F, Kristiansson J, Hård af Segerstad M, Ricksten SE, Nellgård B. Validation of the Nottingham Hip Fracture Score (NHFS) for the prediction of 30-day mortality in a Swedish cohort of hip fractures. Acta Anaesthesiol Scand. 2021;65(10):1413–20.

22. Eschweiler GW, Czornik M, Herrmann ML, Knauer YP, Forkavets O, von Arnim CAF, u. a. Presurgical Screening Improves Risk Prediction for Delirium in Elective Surgery of Older Patients: The PAWEL RISK Study. Front Aging Neurosci [Internet]. 2021 [zitiert 10. März 2022];13. Verfügbar unter: https://www.frontiersin.org/article/10.3389/fnagi.2021.679933

23. Kuhn-Thiel AM, Weiß C, Wehling M, FORTA authors/expert panel members. Consensus validation of the FORTA (Fit fOR The Aged) List: a clinical tool for increasing the appropriateness of pharmacotherapy in the elderly. Drugs Aging. Februar 2014;31(2):131–40.
