# Supplementary material for: Supporting SURgery with GEriatric Co-Management and AI (SURGE-Ahead): A study protocol for the development of a digital geriatrician
Source: PLoS One. 2023 Jun 16;18(6):e0287230. doi: 10.1371/journal.pone.0287230 (PMC10275448; doi:10.1371/journal.pone.0287230)
Supplement: S6 File — (PDF) [file pone.0287230.s006.pdf]

STUDY PROTOCOL

SURGE-Ahead Observation and AI Development Study (OKIE)

April 2023

I hereby confirm the accuracy of the protocol

____________________________________ ____

Date, location

Signature
Prof. Dr. Michael Denkinger, Study Lead

Institute for Geriatric Research, Ulm University Hospital
AGAPLESION Bethesda Clinic, Zollernring 26, 89073 Ulm, Germany

Content

Content 2

1 General Overview 4

1.1 Key points 4

1.2 Summary 4

2 Responsibilities 6

2.1 Participating Institutions 6

2.2 Study Lead 6

2.3 Participating Persons 6

2.4 Additional Partners 7

3 Rationale 9

3.1 Background 9

3.2 Rationale for the study to be conducted 9

3.3 Risk-benefit analysis 10

4 Study goals 11

5 Study design 12

5.1 Study design 12

5.2 Study population 12

5.3 Calculation of sample size 13

6 Study plan 14

6.1 Study duration 14

6.2 Study preparation 14

6.3 Recruitment of the test persons 14

6.4 Recruitment of cognitively impaired subjects 15

6.6 Examination times 16

7 Data management 19

7.1 Data set and questionnaire 19

7.1.1 Existing data sources 21

7.1.2 Assessments and questionnaires 21

7.1.3 Geriatric expert assessment for the discharge destination ... 22
7.1.4 Time-independent review of the patient file ... 22
7.1.5 Activity measurement with the Axivity AX6® sensor ... 22
7.2 Data acquisition ... 23
7.3 Data evaluation ... 24
7.3.1 Programming the AI for the follow-up recommendation ... 24
7.3.2 Description of the current standard of care ... 25
7.3.3 Completing the data set ... 25
7.3.4 Methodological adjustments ... 26
8 Adverse events (AE) / serious adverse events (SAE) ... 27
8.1 (S)AE for participants ... 27
8.2 Adverse events that jeopardize the success of the study ... 27
9 Ethical and legal aspects ... 29
9.1 Consent ... 29
9.2 Costs and compensation for participants ... 29
9.3 Risks for participants ... 29
9.4 Benefit ... 30
9.5 Insurance ... 30
9.6 Data protection concept ... 30
9.6.1 Pseudonymization of the study participants ... 30
9.6.2 Data acquisition via the input mask ... 31
9.6.3 Data acquisition by the AX6® sensors ... 31
9.6.4 Retention and archiving of data ... 31
9.6.5 Data protection and duty of confidentiality ... 31
Literature ... 33

1 General Overview

1.1 Key points

- Prospective observational study over 12-15 months (9 months recruitment (12 months if necessary), 3 months follow-up) at three hospitals of Ulm University Hospital (Department of Trauma, Hand, Plastic and Reconstructive Surgery (UCH), General and Visceral Surgery (AVC), Urology and Pediatric Urology (URO)).
- Evaluation of physical, functional, social, and psychological parameters relevant to geriatric co-management and follow-up decisions in the inpatient setting.
- Use of the collected data in the further course of the project to program an artificial intelligence (AI) that generates a proposal for the best possible aftercare facility and to analyze the current standard of care.

1.2 Summary

**Population**	Patients hospitalized for surgery ≥ 70 years of age with an ISAR[1] score ≥ 2
**Study size**	N = 170 - 240 (incl. 20% dropouts, 3 clinics: 120-190 UCH, 25 AVC, 25 URO)
**Study design**	Prospective observational study
**Duration**	12-15 months (9 months recruitment (12 months if necessary), 3 months follow-up); Start: 01.02.2023
**Goal**	Collection of a data set<br>- For training the AI for the follow-up recommendation of the dashboard.<br>- As a comparison cohort for the intervention study planned in the course (SURGE-Ahead years 4-6) (=representation of the current standard of care).
**Outcomes**	<u>Primary:</u><br>Expert judgment for best follow-up option at discharge & verification at follow-up. The following follow-up options will be recorded:<br>- Geriatric acute care clinic<br>- Specialty or geriatric rehabilitation clinic (inpatient or outpatient)<br>- Home (with or without staff assistance)<br>- Nursing home<br><u>Secondary:</u>

[1] ISAR: Identifying Seniors at Risk

	- Independence in activities of daily living and need for nursing care at discharge and after 3 months<br>- Adverse events and complications during inpatient stay and in the first 90 days after discharge<br>- Medication review at discharge and after 3 months<br>- Standardized assessment of medical discharge reports<br>- Cognition during inpatient stay and after 3 months<br>- Mobility during inpatient stay and after 3 months<br>- Readmission rate within 3 months after discharge<br>- Quality of life of participants after 3 months<br>- Health economic analysis of health services used up to three months after discharge

2 Responsibilities

2.1 Participating Institutions

- Institute for Geriatric Research, Ulm University Hospital
- AGAPLESION Bethesda Clinic Ulm
- Institute for Medical Systems Biology, University of Ulm
- Institute for History, Theory and Ethics of Medicine, University of Ulm
- Department of Psychiatry and Psychotherapy II, Section of Health Economics and Health Services Research, Ulm University Hospital
- Clinic for Trauma, Hand, Plastic and Reconstructive Surgery, University Hospital Ulm
- Clinic for General and Visceral Surgery, Ulm University Hospital
- Clinic for Urology and Pediatric Urology, Ulm University Hospital

2.2 Study Lead

Prof. Dr. Michael Denkinger

Medical Director, AGAPLESION Bethesda Clinic Ulm

Head of the Institute for Geriatric Research, Ulm University Hospital

Mail: michael.denkinger@agaplesion.de; Phone: +49 (0)731 187-184

2.3 Participating Persons

Institute for Geriatric Research University Hospital Ulm and AGAPLESION Bethesda Clinic

- PD Ph.D. Dr. Dhayana Dallmeier: Head of Research Department AGAPLESION Bethesda Clinic Ulm
- Dr. Christoph Leinert: Consultant Physician AGAPLESION Bethesda Clinic Ulm, Research Associate Institute for Geriatric Research
- Dr. Thomas Kocar: Senior Physician AGAPLESION Bethesda Clinic Ulm, Research Associate Institute for Geriatric Research
- Marina Fotteler: Research Associate
- Genia Decker: Medical documentalist
- Gabriele Müller: Senior study nurse,
- Cornelia Heth: Study nurse

Institute for Medical Systems Biology, University of Ulm

- Prof. Dr. Hans A. Kestler: Head of the Institute of Medical Systems Biology
- Dr. Dennis Wolf: Research Associate

Institute for History, Theory and Ethics of Medicine, University of Ulm

- Prof. Dr. Florian Steger: Director of the Institute for History, Theory and Ethics of Medicine
- Dr. Marcin Orzechowski: Research Associate

Department of Psychiatry and Psychotherapy II, Section of Health Economics and Health Services Research, Ulm University Hospital

- Prof. Dr. Reinhold Kilian: Head of the Section for Health Economics and Health Services Research
- Dr. Annabel S. Müller-Stierlin: Research Associate

Clinic for Trauma, Hand, Plastic and Reconstructive Surgery (UCH)

- Prof. Dr. Florian Gebhard: Medical Director
- PD Dr. Konrad Schütze: Consultant Surgeon
- Dr. Adriane Uihlein: Senior Surgeon
- Dr. Raffael Cintean: Senior Surgeon
- Dr. Carlos Pankratz: Assistant Surgeon
- Sibylle Beck: study nurse
- Esther Blaum: study nurse

Clinic for General and Visceral Surgery (AVC)

- Prof. Dr. Christoph Michalski: Medical Director
- Prof. Dr. André Mihaljevic: Consultant Surgeon
- Nadir Nasir: Assistant Surgeon
- Vytautas Stasiunaitis: Assistant Surgeon
- Colette Dörr-Harim: Head of the study center
- Karen Clauss: Study nurse

Clinic for Urology and Pediatric Urology (URO)

- Prof. Dr. Christian Bolenz: Medical Director
- Dr. Felix Wezel: Consultant Surgeon
- PD Dr. Friedemann Zengerling: Consultatnt Surgeon
- Fabia Mangold: Assistant Surgeon
- Vanessa Disque: Study nurse
- Carmen Veliz-Torrico: study nurse

2.4 Additional Partners

Optimedis AG, Hamburg

Pascal Wendel: Head of Health Data Analysis and IT

Section of Health Services Research and Rehabilitation Research (SEVERA), Institute of Medical Biometry and Statistics (IMBI), University Medical Center Freiburg, Germany

Dr. Sebastian Voigt-Radloff: Head of research area health care research with focus on therapy and nursing sciences

AOK Baden-Württemberg, Department of Care Innovation

Anna-Lena Flagmeier: Project coordination

Neu-Ulm University of Applied Sciences (HNU)

- Prof. Dr. Walter Swoboda, Research Professor and Head of Institute DigiHealth
- Prof. Dr. Johannes Schobel, Research Professor Digital Medicine and Care

3 Rationale

3.1 Background

Geriatric co-management of surgical patients can improve treatment, reduce the severity of long-term sequelae, and reduce mortality (1-3). The integration of geriatric expertise has been particularly successful so far in trauma surgery (4). But also in other surgical specialties, such as general surgery (5) or urology (6) patients can benefit from geriatric co-management.

A core aspect of the geriatric treatment approach is the holistic view of patients through a comprehensive geriatric assessment (CGA) performed by a multidisciplinary team. In a CGA, different domains are considered in order to identify physical, psychological, social, and functional limitations and to include them in the treatment (7,8).

However, in view of the increasing number of geriatric patients and the shortage of trained geriatricians, geriatric co-management has not yet been established on a widespread basis, despite its advantages. A particular challenge in the care of geriatric patients is the assessment of the optimal follow-up path (e.g., discharge to home, to a nursing home, to a geriatric rehabilitation clinic or to a geriatric acute care clinic). The SURGE-Ahead project will develop a digital application (dashboard) for improving geriatric co-management in surgical hospitals. Instead of a CGA, a dataset will be defined that maps the minimum requirements for successful geriatric co-management (Minimum Geriatric Dataset - MGDS). Based on the MGDS, the dashboard will display suggestions for 1) evidence-based treatment options of typical geriatric diseases and syndromes based on simple algorithms and 2) suggestions for optimal follow-up care based on artificial intelligence (AI). The dashboard will provide geriatric expertise in surgical clinics. In operation, the system is designed to support the entire multidisciplinary team by providing an initial assessment and treatment recommendations. The goal is to sustainably improve treatment and continuing care for older patients.

SURGE-Ahead was launched in July 2021. The first three years of the project are dedicated to the development of the dashboard. Based on evidence and expert consensus, the MGDS will be defined for the operation of the dashboard. The MGDS is composed of 1) pre- and post-operative assessments and questionnaires (see Section 7.1), 2) existing data from the hospital and laboratory information systems, and 3) data on movement and mobility parameters collected via a body sensor (Axivity AX6).

3.2 Rationale for the study to be conducted

In the planned observation and AI development study (OKIE), the data for the training and development of the AI will be collected in three clinics of the University Hospital Ulm (trauma, hand, plastic and reconstructive surgery, general and visceral surgery, urology, and pediatric urology) with

170-240 patients. With this component, the development of the dashboard can be finalized. The OKIE will thus serve to finalize program development and prepare for an intervention study in subsequent years.

3.3 Risk-benefit analysis

The assessments to be carried out mean an additional effort of just under two hours for study participants. This time is divided into preoperative and postoperative assessments as well as a 30-minute telephone follow-up approximately 90 days after discharge. There is no risk that the assessments will exceed the risks of the patients' current individual hospital treatment. If the survey is a burden for the patient, it will be interrupted immediately and continued at a later time, if necessary. All participants can withdraw from the study at any time and without giving reasons. The assessments are performed by trained personnel (study nurses, study physicians). During the inpatient study phase, the study physicians and study nurses s are available to answer questions.

If desired, the knowledge gained can be passed on to the test persons after the study has been completed. In addition, the test persons make a valuable contribution to the scientific knowledge gained.

4 Study goals

During the OKIE, the data set defined for SURGE-Ahead (MGDS) will be collected for 170 to 240 patients at the three participating hospitals. In addition to the MGDS, a physician experienced in geriatrics will document a recommendation for a follow-up option for all subjects before discharge from the hospital based on the collected MGDS data, the medical record, and a personal patient contact. This recommendation is verified again at follow-up, corrected if necessary, and established as the gold standard. This is used to train the AI to generate a suggestion for the best possible discharge destination in the final dashboard.

Thus, the primary goal of OKIE is to collect a dataset for training the AI to generate a recommendation for a post-acute care facility.

The secondary goal is to use the OKIE data set as a comparison cohort for the intervention study planned during the course (SURGE-Ahead year 4-6) with the completed dashboard (=representation of the current standard of care).

5 Study design

5.1 Study design

A **prospective observational study with a follow-up after three months** will be conducted in the clinics for trauma, hand, plastic and reconstructive surgery, general and visceral surgery and urology and pediatric urology of the University Hospital Ulm. There is no intervention, so there is no active involvement in the regular care delivery during the study.

Important data for the treatment and for the evaluation of the treatment success of geriatric patients will be collected at different assessment points and during the follow-up (MGDS). Based on the collected MGDS data, the medical record and a personal patient contact, a physician experienced in geriatrics from the project team will also document an expert assessment for an optimal follow-up option for all subjects. This assessment will be verified again at follow-up by the same geriatrician and will serve as a reference for the training of the AI. The assessment of the geriatrician is not shown to the treating clinical staff and has no influence on the continuation of care of the subjects.

According to the study design, blinding of the study team is not possible and not necessary since no intervention takes place.

5.2 Study population

Inclusion criteria

- Patients ≥ 70 years of age admitted for an inpatient stay with surgical intervention in one of the three participating hospitals and whose surgery has not yet been performed (emergency or elective admissions).
- Patients with an ISAR score ≥ 2 (9,10).

Exclusion criteria

- Patients in a palliative treatment situation (life expectancy < 3 months based on clinical assessment by the treating physician).
- Patients who are incapable of giving consent and for whom no legal guardian or authorized person is available.
- Collection of assessments not possible due to limited ability to communicate (e.g., due to lack of language skills).
- Patients already participating in another study.
- Patients with a presumed length of stay of < 3 nights.

5.3 Calculation of sample size

A larger dataset improves the performance of the AI being developed. If the dataset is too small, there is a risk that the AI will not be adequately trained, but will make the decision largely by chance (11). Starting from a minimum number, a recruitment corridor is therefore aimed at, which can be exhausted if recruitment progresses well. With an expected dropout rate of 20%, 170 - 240 patients are to be recruited. The focus here will be on the Department of Trauma, Hand, Plastic and Reconstructive Surgery, as this is where the greatest range and variability of possible follow-up options is covered, and patients treated here are likely to have the greatest benefit from a subsequent dashboard application. The following breakdown is targeted (may be slightly adjusted as the study progresses):

- UCH: 120 - 190 subjects.
- AVC: 25 subjects
- URO: 25 subjects

Based on ±1000/year of inpatients ≥ 70 years of age in the Department of Trauma, Hand, Plastic and Reconstructive Surgery (of whom ±500/year have an ISAR of 2 or higher), ±800/year in the Department of Urology and Pediatric Urology, and ±1400/year in the Department of General and Visceral Surgery, the recruitment numbers appear realistic.

The target case numbers are based on an assessment of feasibility regarding AI training and recruitment, no explicit case number calculation was performed.

6 Study plan

6.1 Study duration

The study is planned for 12 months from Feb 1, 2023, to Jan 31, 2024, with a recruitment period of nine months and a follow-up of three months. If the recruitment goal is not reached within the nine months, the recruitment period can be extended by another three months. In this case, the study will run with follow-up until 30.04.2024 at the latest.

6.2 Study preparation

The study will be prepared between Dec 1, 2022, and Dec 31, 2022. The study nurses, physicians, nursing staff and the data manager will be briefed on the study and the study procedure. At least one information session will be held per clinic, during which there will also be an opportunity to ask questions. The aim is to prepare the local clinical staff as well as possible for the start of the study to gain their support in the selection and recruitment of suitable patients.

On site in the clinics, the study nurses are the contact persons for acute questions. Here, care is taken to ensure as comprehensive a presence as possible. In addition, physicians are available in all three clinics (see chapter 2.3), who are also part of the SURGE-Ahead study team.

6.3 Recruitment of the test persons

Screening with the ISAR score is an official recommendation and is well known in the centers. In most cases, the score is collected as standard at admission. For the OKIE, all patients 70 years of age and older who are admitted to UCH, AVC or URO for an inpatient surgical procedure will be screened with the ISAR. Recruitment of potential subjects will occur after confirmation of inclusion criteria. The verification of the inclusion criteria is paper based in favor of an easier patient contact. After inclusion, the data are subsequently recorded electronically (see also section 7.2).

Potential subjects will be informed about the possibility of participating in the SURGE-Ahead project immediately after hospital admission or, in the case of elective surgery, during a preliminary discussion. In case of interest, the patients will be informed orally and in writing by the study physicians about the aims, procedures, risks, and data protection of the research project and will receive written patient information. This also includes information about the use of a lumbar sensor (company Axivity AX6®) for the collection of postoperative mobility data during the inpatient stay.

All patients have the opportunity to ask questions during the information session. Consent to participate in the study is given verbally and in writing and is voluntary.

If the respondents are too stressed by the situation due to their underlying disease (e.g., fracture) and/or are unable or unwilling to continue the interview for other reasons, they can interrupt the interview at any time. In this case, it is also possible for some data to be collected by a caregiver (e.g., relatives, friends) via external anamnesis.

In the follow-up survey (T6), the respondent's family doctor will also be asked about the quality of follow-up care. To enable the questioning of relatives and family physicians, the test persons give a release of confidentiality. The required contact data for relatives and general practitioners are first recorded in the release from confidentiality and later transferred to the paper-based identification list together with the respondent's identification number. The release from confidentiality is requested upon enrollment in the study but can also be signed during study participation. However, without a signed release of confidentiality, no survey of the primary care physician can be conducted during the follow-up.

After patient consent, the patient is enrolled in the study. For documentation of patient selection and recruitment progress, an anonymized screening list is maintained in all centers, including any reasons for exclusion.

6.4 Recruitment of cognitively impaired subjects

According to the Declaration of Helsinki as amended in October 2013 (taking into account points 28.-30.), the inclusion of non-consenting individuals in medical studies is only permissible under narrow conditions (12). These provide that the patient 1) either derive a direct benefit of their own from participating in the study, or 2) the group of patients that this individual represents is likely to benefit from the findings of the study and the study presents minimal risks and burdens. In the case of SURGE-Ahead, case 2) applies. Current evidence from the Geriatric Traumatology Center (ATZ) Ulm demonstrates that the group of cognitively impaired patients is particularly vulnerable to perioperative complications, mortality, or persistent functional deficits (13). They represent about 40% of the patients cared for. Thus, this group would particularly benefit from geriatric co-management and optimal follow-up decisions.

If the patient is found to be incapable of giving consent during the information session, information and consent will be obtained from the legal guardian or proxy.

If a patient is incapable of giving consent and no legal guardians or proxies are available, participation in the study is not possible (exclusion criterion). Also, when the power of attorney or care directive cannot be presented, study participation is not possible. Figure 1 shows the recruitment procedure for patients with cognitive impairment.

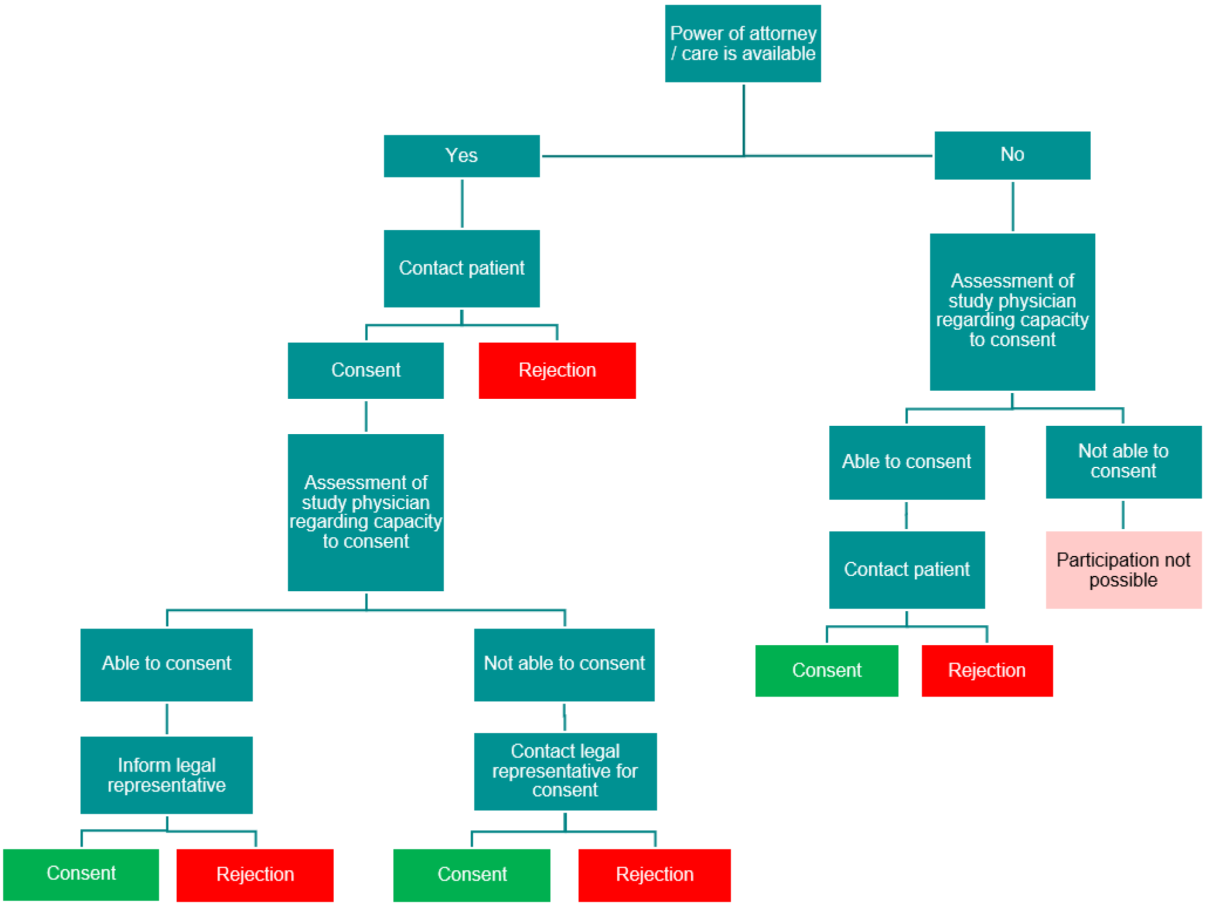

Figure 1: Consent algorithm in patients with cognitive impairment.

6.6 Examination times

There will be up to seven data collection time points, T0-T6, depending on the length of stay:

- T0: Preoperative
    - T0.1: Assessments to be collected preoperatively
    - T0.2: Assessments to be collected pre- or postoperatively (depending on the clinical situation of the subjects)
- T1-T4: Postoperative inpatient days 1, 3, 5, and 7 after surgery (shorter length of stay may require fewer assessment time points).
- T5: 1-2 days before discharge
- T6: Follow-up 90 days after discharge (± 7 days).

In addition, there are surveys that are conducted after discharge, but are not tied to a fixed time period (Figure 2).

After inclusion of the subjects, the preoperative assessments (T0) are performed (see Chapter 7). Some assessments are performed preoperatively for all subjects (T0.1). These assessments can be performed

0-3 days before surgery. If surgery is postponed for more than three days, the preoperative assessments must be repeated. To consider the possibly lower resilience of participants preoperatively, especially considering the longer assessment section T0.2, it is possible to collect a part of the preoperatively planned assessments, if necessary, postoperatively or through an external history. On days 1, 3, 5 and 7 after surgery, the postoperative interviews and assessments will be performed (T1-T4). Here, the study nurse first looks at the patient's file and the system data (see below) and records relevant data. Subsequently, the interview with the patients takes place. On day 1 after surgery, the mobility sensor (Axivitiy AX6®) is attached (see chapter 7.1.5). If patients are discharged before day 7 after surgery, the postoperative assessments will only be performed until the discharge day. At discharge, the system data will be recorded again by the study nurse. In addition, 0-3 days before discharge, the expert assessment will be performed by the geriatric experienced physician based on the collected MGDS data, the medical record and a personal patient contact including performance of the discharge assessments (T5). This assessment is used exclusively for the training of the AI and has no influence on the actual treatment of the patient. Following discharge, a retrospective recording of adverse events and complications, as well as a critical assessment of the medical discharge report regarding completeness and quality (time-independent) is carried out.

Parallel to the assessments, which are completed together with participants, data from the hospital information system (HIS), the patient file, and the laboratory information system (LIS) are recorded manually. The master data from the HIS are recorded once. Clinical HIS data (e.g., medication, diagnoses) and the data from the LIS are recorded preoperatively and on days 1, 3, 5 and 7 after surgery and at discharge, or checked for new entries and updated (T1-T5).

90 days ($\pm$ 7 days) after discharge, a telephone follow-up (FU) is conducted (T6). This takes about 30-40 minutes (see chapter 7). In the case of patients who are not capable of giving consent, the caregiver is preferably interviewed. However, if this person is not a member of the family (professional caregiver), the patient is interviewed. In addition, an attempt is made to obtain the assessment of the family doctor. Figure 2 shows an overview of the survey times.

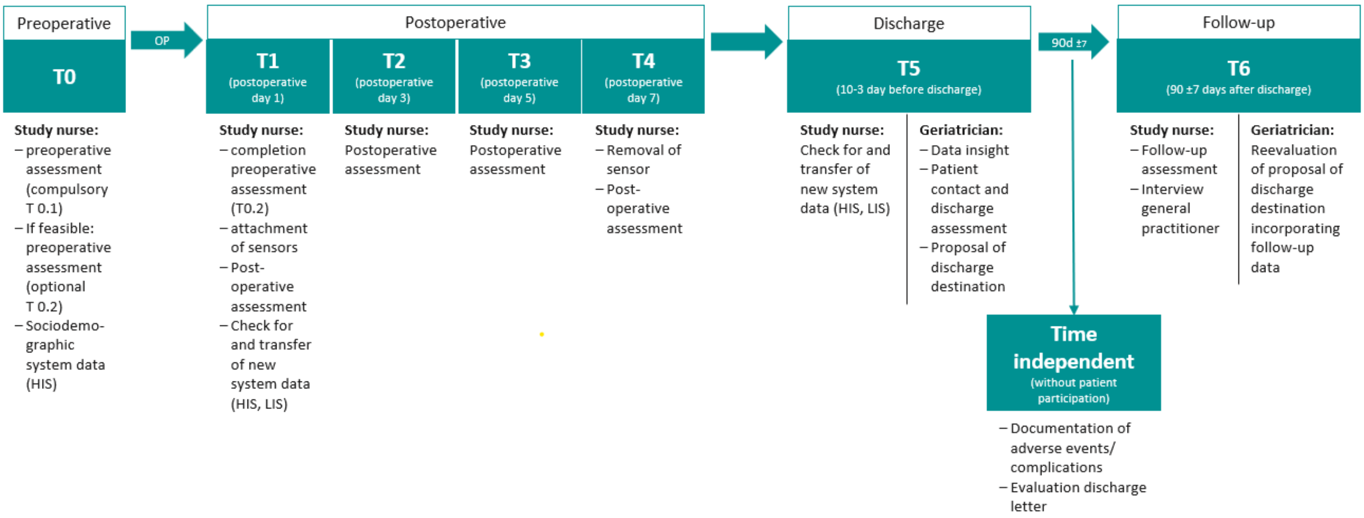

Figure 2: Overview of the examination times

7 Data management

7.1 Data set and questionnaire

For the development and functionality of the dashboard, a data set, the MGDS, was defined. It consists of parameters that are important for the treatment of geriatric patients. The parameters are based on published scientific findings and clinical guidelines derived from them. The literature was reviewed as part of systematic literature reviews (14,15). The selection of data and assessments was also preceded by several workshops (11/2021 - 05/2022) with SURGE-Ahead collaborators and an international advisory board.

The MGDS consists of 1) parameters extracted from existing systems or files (hospital information system, laboratory information system, anesthesia sheet, patient file), 2) data collected by means of validated assessments and questionnaires, and 3) sensor data on mobility aspects. In addition, the assessment of the physician experienced in geriatric medicine is also collected as part of the OKIE. Table 2 lists all data, examination times and possible sources.

For the assessments and questions with patient contact listed in Table 2, we assume a total time expenditure < 2 h (distributed over the individual survey time points). This estimate is largely based on the measurement results from the SURGE-Ahead pretest (Ethics Application No. 170/22 - Du/Sta, decision dated May 30, 2022) as well as experience from the literature and personal estimates. The approximate expected effort for participants for each survey time point is also shown in Table 2.

Table 2: Overview of the data to be collected and assessments to be performed, incl. the examination times

Domain	Assessment/ Questions	Time of examination(s)							
		T0.1	T0.2	T1	T2	T3	T4	T5	T6
**Assessments with patient contact**									
Expected time expenditure for participants (in min)		6	30	12	7	7	8	5	40
**Preoperative assessments with the participant:in**									
Screening geriatric patients	ISAR (Identifying Seniors at Risk) *#	x							
Pain	NRS-P (Numeric Rating Scale - Pain) #	x		x	x	x	x		x
Activities of daily living (ADL)	Barthel Index *#	x [1]		x	x	x	x		x
Delir	4AT	x		x	x	x	x		
**Pre- or postoperative assessments with the participant:in (depending on the clinical situation).**									
Sociodemographics	3 questions *#		x	(x)					
Social history	11 questions *#		x	(x)					
Self-Related Health	5 questions*		x	(x)					x
Patient-centered goals	4 questions *#		x	(x)					

Cognition	MoCa 5-min (Montreal Cognitive Assessment Test)		x	(x)					x
Depression	PHQ-4 (Patient Health Questionnaire)		x^2	$(x)^2$					x^2
Malnutrition	NRS-N (Nutrition Risk Score Screen) *#		x	(x)					x
Dysphagia	1 question *		x	(x)					
Smoking	1 question *#		x	(x)					
Multimedication	2 questions *		x	(x)					x
Sensory (vision/hearing)	3 questions *		x	(x)					
Urinary incontinence, bladder catheter	2 questions *#		x	(x)					x
Falls	3 questions *		x	(x)					
Quality of life	EQ 5D 5L, EQ5 VAS		x	(x)					x
Mobility	New Mobility Score *		x^2	$(x)^2$					x
	Charité Mobility Index (CHARMI) #		x^2	x	x	x	x		x^3
	Sensor data			x<br>continuous T1 - T4					
Frailty	CFS (Clinical Frailty Scale)		x^2	$(x)^2$				x^2	
Geriatric Expert:in recommendation *(may be performed several times in case of long inpatient stays).*	2 questions							x	
**To be completed by some participants (n=30)**									
Movement diary				x<br>continuous T1 - T4					
**Telephone questionnaire follow-up after 90d**									
Social history Follow-up	9 questions *								x
Medical course Follow-up	8 questions *#								x
Health economics and med. services	CSSRI *								x
Questions for family doctor	4 questions *								x
Assessment geriatrician:in follow-up	3 questions								x
**Data primarily from the patient file**									
Details inpatient stay	16 Questions/ Data #		x<br>(to be completed during the study)						
Vital signs	Blood pressure, pulse, oxygen saturation, temperature #			x	x	x	x		
Comorbidities 1 *(OP data are also recorded for re-operation(s)).*	Diagnoses from admission letter Surgery and<br>Anesthesia sheet incl. #<br>- Weight/Size<br>- ASA<br>- OP urgency		x	(x)					

	- Alcohol consumption<br>- Comorbidities								
Comorbidities 2	Query of 15 disease patterns *#		x	(x)					
Anthropometry	Weight, height; BMI *#		x	(x)					x
Wounds/Decubiti	2 questions *#							x	x
Laboratory data of the inpatient stay #	Haemoglobin #							x	
	MCV #							x	
	Leukocytes #							x	
	CRP #							x	
	Creatinine #							x	
	eGFR n Cockcroft-Gault #							x	
	Sodium #							x	
	Potassium #							x	
	Albumin #							x	
	Multi-resistant germs #							x	
Medication #	According to patient curve							x	x
General information on discharge	Discharge target 1 question #							x	
Time-independent data collection from patient file									
Adverse events and complications at discharge	Global Trigger Tool (GTT)							x	
Assessment discharge letters	9 questions #							x	

[1]Retrospective (approx. 2 weeks before recording) and current state
[2]Retrospective (approx. 2 weeks before recording)
[3]To be collected as Patient Reported Outcome (CHARMI-PROM).
*If necessary, to be collected by relatives or caregivers via external anamnesis (with signed release from confidentiality)
#If necessary, to be collected from the patient's file.

7.1.1 Existing data sources

Many data, such as information on weight, height, decubiti, adverse events and complications, medication (incl. frequency and dose), alcohol/smoking behavior, and laboratory parameters are already routinely collected and documented at different locations/in different systems. These sources will be reviewed first to minimize the burden on the subject. Explicitly, the anesthesia sheet, the patient chart, the medication schedule, the electronic patient record in the hospital information system (HIS), and the laboratory information system (LIS) will be reviewed. If the data cannot be extracted from existing sources, the subjects will be asked. The source of the data is documented in all cases.

7.1.2 Assessments and questionnaires

The biggest part of the MGDS consists of clinical assessments covering the most important domains of geriatric patient care (e.g., cognition, mobility, pain, continence, activities of daily living or comorbidities). In addition, the German version of the "Client Sociodemographic and Service Receipt Inventory (CSSRI)" (16,17) (adapted to project requirements) will be used at follow-up T6 to capture

health service utilization for the health economic evaluation. Some assessments and questions will be collected multiple times to allow for progress documentation. If necessary, individual assessments and questions can also be collected from relatives via an external history (see Table 2).

7.1.3 Geriatric expert assessment for the discharge destination

The expert recommendation by a physician experienced in geriatrics is made at T5 (discharge) and at T6 (follow-up). In this context, a recommendation for the best possible follow-up option is documented based on the assessment data of the MGDS collected at discharge from the acute hospital, the medical record, and a personal interview with the subjects (no interaction with the clinical treatment team takes place here). This recommendation is then reconfirmed or adjusted at the follow-up appointment based on retrospective observation of the further course. In case of long duration or acute deterioration after T5, the expert assessment may be repeated.

7.1.4 Time-independent review of the patient file

The needs of geriatric patients are often complex and interdisciplinary. To analyze the course of inpatient treatment of the participating subjects regarding the need for geriatric co-management, various contents of the patient file will be critically analyzed. This analysis will be carried out retrospectively and independent of the inpatient treatment of the test persons.

1. Evaluation of medical discharge reports: The medical discharge report (physician's letter) is the most important documentation tool for presenting the quintessence of the inpatient treatment course and ensuring seamless continuation of medical care in a continuing care setting. It represents an essential means of communication between different sectors of the healthcare system and is an important aspect of a successful continuity of care (COC). To demonstrate the quality of the content of the discharge reports, a standardized evaluation with a scoring system is carried out during the course of the study (18).
2. Adverse events during treatment: Due to their multimorbidity and frailty, geriatric patients are particularly vulnerable to adverse events (AEs). To represent the rate of surgical and nonsurgical AEs and complications, these are collected using a retrospective chart review based on the Global Trigger Tool (GTT) (19).

7.1.5 Activity measurement with the Axivity AX6® sensor

Mobility and physical activity are essential prerequisites for quality of life, independent care, and independent living in old age. Immobility, on the other hand, represents an important cause of disability, often leading to need for care and dependence in old age. To better understand physical activity and mobility in the context of surgery, in addition to the assessments, an activity measurement will be performed using a six-axis accelerometer (AX6®, Fa. Axivity Ltd., Newcastle, United Kingdom) attached to the lower back (lumbar) (see Appendix: Data Sheet AX6). The sensor has an official

approval and a CE-certificate and has already been used at the Institute for Geriatric Research in other projects with geriatric patients without side effects (e.g.: Prometheus study approved by the ethics committee of the University of Ulm (ethics vote no. 26/21 / year 2021)).

The sensor is attached on day 1 (T1) after surgery with two waterproof foils (OPSITE FLEXIFI PNZ: 07478029) at the level of the waist (lumbar vertebra 5) in the middle of the back (see Fig. 3). The height of attachment is documented (heel to upper edge of sensor; measurement while lying down). Body hygiene (washing, showering) is still possible without restrictions. If the sensor cannot be attached at T1 due to the patient's physical condition, it can also be attached to the thigh from T2 onwards. Attachment to the thigh is also possible if the participants refuse the sensor on the spine. The sensor is worn for up to 7 days, depending on the duration of the inpatient stay and the day of attachment, and is removed at T4 at the latest. The measurement is always started at the beginning of a full day and ended at the end of a full day. It thus covers up to five complete days. No measurement is performed on the incomplete days of attachment and removal. Data recorded by the AX6 include, for example, the number of steps per day, the duration of lying, standing, and walking times in min per day, and the number of lying-sitting and sitting-standing movements.

Among others, the freely available GGIR package (Raw Accelerometer Data Analysis) for R is used for the analysis (20). To test these and possibly other algorithms for the OKIE patient collective, 30 participants (N=10 per recruiting clinic) will complete a movement diary while wearing the sensor (T1 - T4). Cognitively impaired participants are excluded from this. Here, among other things, the times of getting up in the morning and going to bed in the evening are recorded.

7.2 Data acquisition

Data collection is performed by study nurses. One position per clinic is planned for this purpose (they should also support each other). All three clinics and the study nurses will be equipped with a 2in1 device (laptop and tablet) from Dell for data collection. The device is operated in the secure hospital network and has access to the HIS and LIS. It is operated in cooperation with the Center for Information and Communication of the University Hospital Ulm and meets the strict IT requirements for operation in the network of the University Hospital Ulm.

A dedicated data entry application was programmed for OKIE, which is hosted within the network of the University Hospital Ulm and can be accessed via any standard browser (e.g., Edge, Chrome, Firefox) within the network. For data entry, the study nurses call up the application in their browser via a secure connection and dial into the interface using their individually assigned access data. The data are entered by the study nurses using preset input masks adapted to the standardized questionnaires (see Fig. 4). Data are entered using the mouse and keyboard or the touchscreen of the device. Data that

can be collected without the participation of the subjects or their relatives (i.e., data from the HIS or LIS) are extracted by the study nurses at a workstation in the University Hospital Ulm and entered into the input mask. Before each assessment, the conditions during the interview and the isolation status of the patient are recorded. All data entered via the input mask are stored in a password-protected database located on the same server as the application.

Regular project monitoring is used to continuously monitor the progress of the project. Data quality is also checked to identify problems in good time and to be able to take countermeasures.

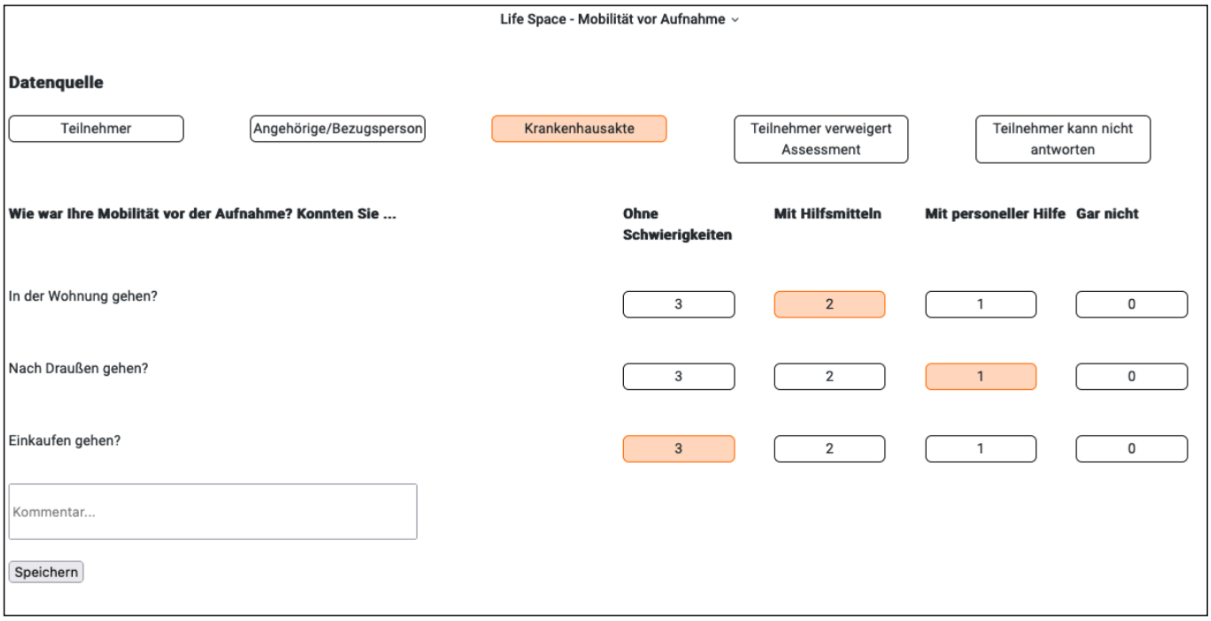

Figure 4: Example of the view of the study participant during data entry (here: New Mobility Score)

7.3 Data evaluation

Primarily, the goal of the current OKIE is to collect a training data set for the AI. In years 4-6 of the SURGE Ahead project, an intervention study is planned to test the dashboard in clinical practice. Therefore, the OKIE will also serve as preparation as well as a comparison cohort for the evaluation of the intervention. Key outcome parameters are listed below.

7.3.1 Programming the AI for the follow-up recommendation

The goal of the entire SURGE-Ahead project is to develop a digital application that maps perioperative geriatric co-management and makes a recommendation for appropriate follow-up care facilities. For this second aspect, we currently assume the following main categories (classes) in the German healthcare system:

- Geriatric acute care clinic
- Rehab facility
    - Specialized rehabilitation facility
    - Inpatient geriatric rehabilitation facility (mainly).

    - Outpatient geriatric rehabilitation facility (hardly any)
- Nursing home
- Home
    - without personnel help
    - with personnel help

In addition, a few patients may be transferred to another acute care hospital, geriatric psychiatric facility, or hospice. Because the widest range of these follow-up options is utilized in the Department of Trauma, Hand, Plastic, and Reconstructive Surgery, these patients are overweighted in recruitment. In general and visceral surgery and urology, project partners estimate that approximately 70-90% of patients are discharged home.

To predict the discharge destination as accurately as possible, sufficient data points are required for each COC destination (class). The exact number of data points needed in total and per class varies with the model used (e.g., linear and non-linear) and the targeted performance. Since the model is determined post-hoc and exploratory, the goal is to distribute the data points as evenly as possible across classes. A data set is needed in which the categories to be predicted occur often enough (11). Therefore, the initial goal for OKIE is a four-category approach, namely discharge 1) to an acute geriatric hospital, 2) to a rehabilitation hospital, 3) to a nursing home, and 4) to home. If possible, further calculations are then performed with the collected dataset for refinement.

7.3.2 Description of the current standard of care

The OKIE dataset captures the current standard of care and is intended to assess the need for geriatric co-management with respect to various primary and secondary outcomes. Currently, descriptive statistical methods are planned to describe the different endpoints. This also includes health economic considerations. The cost-effectiveness ratio of the resources used will be determined from an economic perspective based on the utilization of health care services (CSSRI) and quality of life (EQ 5D 5L) using the net benefit method. The cost of illness is estimated by multiplying the units of services used by the determined costs of these units, each for a period of 3 months (T0 to T6). For the intervention study planned in a future project phase, the collected data will be used as a comparison cohort, if necessary. For this intervention study, a separate ethics application will be submitted in the course.

7.3.3 Completing the data set

The data collected will be used to calculate cross-cutting scores and verify them with the data so that they can be used in the planned intervention study if necessary. These include, for example, the Nottingham Hip Fracture Score for predicting 30-day mortality after hip fracture. The score is based on the following seven parameters: age, sex, number of comorbidities, cognitive status, preoperative

living situation, hemoglobin concentration at admission, and the presence of malignant tumors (21). Furthermore, using the OKIE data, the delirium risk score and frailty index from the PAWEL study (22) (the research team of the Institute of Geriatric Research was also involved in this study) will be validated in a form adapted for the MGDS.

The drug treatment plan is reviewed regarding over- and under-therapy as well as potentially inadequate medications (PIM) for the elderly. For this purpose, the drug treatment plan is reviewed at the beginning of the inpatient treatment, at its completion, and at the follow-up time using validated tools (including FORTA) (23). Optimedis AG supports the automated calculation of the FORTA score. The analysis is performed using Optimedis software provided in a Docker. Only the study identification number, age, ICD, and ATZ codes are required for the analysis.

7.3.4 Methodological adjustments

Within the framework of the OKIE, it should be examined whether all selected assessment times (especially for the progress assessments) are also necessary in the later intervention study, or whether adjustments should still be made here (e.g., deletion of the assessment time T2 due to missing or only minor change). Further methodological adjustments are also conceivable.

8 Adverse events (AE) / serious adverse events (SAE)

8.1 (S)AE for participants

If AEs or SAEs occur during the interview, the study headquarters will be informed of the incident. Subsequently, it is checked whether there is a connection with the study. If this is the case, the responsible ethics committee will be informed within 7 working days. Optimal therapy and care can be always ensured by the clinical treatment team on site. Furthermore, in case of adverse events, geriatric expertise from the study team can be accessed at any time.

8.2 Adverse events that jeopardize the success of the study

To be prepared for possible contingencies and adverse events that could jeopardize the progress and success of the study, various measures were defined. These are recorded in Table 3.

Table 3: Risks for study success and measures

**Risks**	**Measures**
Recruitment rate too low	<u>Preventive:</u><br>- Information event for clinical staff<br>- Comprehensive briefing of the study nurses<br><u>Reactive:</u><br>- Extension of the recruitment period from 9 to 12 months<br>- Recruitment of patients with an ISAR ≥ 1
Data loss	<u>Preventive:</u><br>Regular local and remote backup of the entire database<br><u>Reactive:</u><br>Restoring the database from backup
Missed examination times (e.g., due to vacation, illness, unavailability of test persons)	- Substitution by other study nurses or in case of emergency by the study director.<br>- If no representation/no interview possible:<br>o Individual progress assessments (T1-T4) can be omitted in case of emergency.<br>o T0, T5 and T6 must be made up if possible (then with corresponding note)
Processes inconsistent between clinics	- At the beginning, regular telephone conference between the project team, the study physicians, and the study nurses.<br>- Regular process monitoring by the study management.<br>- Regular data monitoring by members of the study management team
Drop-outs higher than expected	Increase the number of recruits

Risk of delay of the overall study	Conduct review of patient records and discharge reports with a reduced number of study participants or eliminate them entirely.
Covid-19	Increased hygiene and precautionary measures (see appendix: hygiene concept)

9 Ethical and legal aspects

9.1 Consent

All participants will be informed verbally and in writing about the study objectives, the procedure, possible risks, and the use of the data (see Appendix: Information for patients). Participants will be explicitly informed that they can contact the study team if they have any problems or questions, or that they can also terminate their participation in the study at any time. The contact information of the study staff (telephone number, e-mail address) will be provided to the participants with the educational documents.

Consent is obtained for participation in the study, for the external medical history to be taken by relatives/caregivers and general practitioners (release from obligation to maintain confidentiality), and for the recording of mobility data by the sensor. The participants confirm their consent with their signature during the educational interview.

For patients who are not capable of giving consent, a legal guardian is consulted (see also Chapter 6.4).

9.2 Costs and compensation for participants

There are no costs for the participants. Participants do not receive any monetary or other compensation.

9.3 Risks for participants

The risks for the participants are low. If the interview is too stressful for the patients, it will be interrupted immediately and continued later if necessary. Since we believe that this could happen in the preoperative assessments (especially in the case of emergency patients), only the four most important domains are queried here (identification of geriatric patients (ISAR), delirium (4AT), ADL (Barthel), pain (NRS-P)). The ISAR score is already routinely collected in most cases at Ulm University Hospital. The pain scale and the Barthel index are used in all clinics. Since the ISAR score is part of the screening, usually only the 4AT test is added preoperatively. An additional time expenditure of about two minutes is to be expected for this.

The risk of psychological complaints being caused by participation is considered to be low. If necessary, the longer preoperative assessment period (T0) can be divided into 2 periods (T0.1 and T0.2); of course, a withdrawal from the voluntary participation in the study is possible at any time.

The risk of Sars-Cov2 infection through contact with study personnel is also considered to be low. All study personnel are required to undergo regular testing using rapid antigen tests and to wear an FFP2 mask for the duration of their stay at Ulm University Hospital. The study personnel will also be provided

with sufficient disinfectant. In addition, the University Hospital Ulm has its own, comprehensive hygiene concept, according to which the study personnel must comply. Any adaptations to the hygiene concept of Ulm University Hospital that may take place in the further course of the study (e.g., higher-frequency rapid antigen testing for employees) will also be implemented by the study personnel.

All participants can withdraw from the study at any time and without giving reasons.

9.4 Benefit

If desired, the data collected from and about them can be passed on to the participants after completion of the study. In addition, the test persons make a valuable contribution to the scientific knowledge gained.

During the inpatient study phase, the study physicians and nurses are available to answer questions.

9.5 Insurance

During participation in the study, all subjects are covered by insurance.

The University Hospital Ulm and the staff participating in the study are insured against liability if the subjects suffer damage through their fault.

9.6 Data protection concept

9.6.1 Pseudonymization of the study participants

After the inclusion criteria have been checked and the subjects have given their informed consent, the study nurse creates a new entry in the study database. This automatically generates a study identification number. This number consists of an identification number of the involved department (UCH 11, AVC 12, URO 13) and a consecutive number of the included patients. This results in the following pseudonymization format:

- for UCH: 11001, 11002, ...
- for AVC: 12001, 12002, ...
- for URO: 13001, 13002, ...

No personal data such as name, address, or telephone number are recorded in the study database. To decode the pseudonymized data sets, a study participant identification list with all participating patients is kept in handwriting. The study participant identification list contains the study identification number, full name, date of birth, address, and telephone number. In addition, the contact details of a relative or other caregiver or, in the case of persons who are not capable of giving consent, of the authorized person, as well as the family doctor, are stored if necessary. The date of inclusion and, if applicable, the date of discontinuation, are documented for all subjects. The paper-based

identification list is the only way to assign records in the study database to an individual. The data in the list are used to identify participating patients, to clarify ambiguities or to hand out the recorded data to the subjects at their request. The identification lists remain in the respective clinics in locked cabinets in rooms with restricted access. The identification lists are updated and maintained by the authorized study personnel on site.

9.6.2 Data acquisition via the input mask

Data is collected via a specially programmed user interface. As mentioned in chapter 7.2, communication between the study nurse and the application is encrypted and password protected. The data entered is stored in a password-protected database, from which a regular backup is created. The data remains only within the network of the University Hospital until the study is completed.

9.6.3 Data acquisition by the AX6® sensors

All collected data is stored locally on the memory card of the sensor. At the end of the wearing period, the sensor is removed, and the data read out by the study nurse. The sensor data are initially stored in a separate database, provided with the unique study identification number. Only after the raw data have been evaluated are they fed into the study database. The sensor is hygienically cleaned, loaded, recalibrated and can subsequently be used again in the study.

9.6.4 Retention and archiving of data

The study-related, pseudonymized data remain on the server of the University Hospital Ulm until the end of the study. Afterwards, the data are transferred on a secure data carrier (e.g., USB stick) to the study center at the AGAPLESION Bethesda Clinic Ulm, where they are transferred to the server of the Institute of Geriatric Research and stored in a cloud operated by the institute. This is protected from unauthorized access by a web application firewall (WAF). Access is only possible through 2-factor authentication by authorized employees of the Institute. Data is stored for at least 10 years in accordance with applicable law. Informed consent forms will remain in the respective clinics and will be stored separately from the study data, also for 10 years, in a locked cabinet, in a locked room suitable for data storage. The study participant identification list with the pseudonymization codes of the study participants will also remain in the respective clinic and will be kept securely locked together with the consent forms. The identification lists will be destroyed after completion of the study and complete data cleaning. Data cleaning will be performed by authorized study personnel of the University and the University Hospital Ulm.

9.6.5 Data protection and duty of confidentiality

All employees involved in the project are trained in advance on how to handle the collected study data responsibly and to comply with the data protection guidelines and undertake to do so in writing when they join the project. All medical project staff are subject to medical confidentiality.

Access to the data collection program is only possible with personal identification and password by the employees involved in the data collection of the study and the study management.

Access to the Institute of Geriatric Research server is protected by 2-factor authentication. Only authorized study personnel with personal identification and password have access to the study server. By the assigned role, study personnel can only edit and view the projects or data for which they have authorization.

Literature

1. Rapp K, Becker C, Todd C, Rothenbacher D, Schulz C, König HH, et al. The Association Between Orthogeriatric Co-Management and Mortality Following Hip Fracture. Dtsch Arzteblatt Int Jan 24, 2020;117(4):53-9.

2. Grigoryan KV, Javedan H, Rudolph JL. Ortho-Geriatric Care Models and Outcomes in Hip Fracture Patients: A Systematic Review and Meta-Analysis. J Orthop Trauma. March 2014;28(3):e49-55.

3. Shahrokni A, Tin AL, Sarraf S, Alexander K, Sun S, Kim SJ, et al. Association of Geriatric Comanagement and 90-Day Postoperative Mortality Among Patients Aged 75 Years and Older With Cancer. JAMA Netw Open. Aug 19, 2020;3(8):e209265.

4. Falaschi P, Marsh D, editors. Orthogeriatrics: the management of older patients with fragility fractures [Internet]. Cham: Springer International Publishing; 2021 [cited February 8, 2022]. (Practical Issues in Geriatrics). Verfügbar unter: http://link.springer.com/10.1007/978-3-030-48126-1

5. Kmietowicz Z. Emergency laparotomy: lack of geriatrician input leaves frail patients at double risk of death. BMJ. Nov 13, 2020;371:m4437.

6. Braude P, Goodman A, Elias T, Babic-Illman G, Challacombe B, Harari D, et al. Evaluation and establishment of a ward-based geriatric liaison service for older urological surgical patients: Proactive care of Older People undergoing Surgery (POPS)-Urology. BJU Int July 2017;120(1):123-9.

7. Ellis G, Gardner M, Tsiachristas A, Langhorne P, Burke O, Harwood RH, et al. Comprehensive geriatric assessment for older adults admitted to hospital. Cochrane Database Syst Rev. September 12, 2017;9:CD006211.

8. Eamer G, Taheri A, Chen SS, Daviduck Q, Chambers T, Shi X, et al. Comprehensive geriatric assessment for older people admitted to a surgical service. Cochrane Database Syst Rev. Jan. 31, 2018;1:CD012485.

9. Warburton RN, Parke B, Church W, McCusker J. Identification of seniors at risk: process evaluation of a screening and referral program for patients aged ≥75 in a community hospital emergency department. Int J Health Care Qual Assur. 1 Jan 2004;17(6):339-48.

10. McCusker J, Bellavance F, Cardin S, Trépanier S, Verdon J, Ardman O. Detection of older people at increased risk of adverse health outcomes after an emergency visit: the ISAR screening tool. J Am Geriatr Soc Oct 1999;47(10):1229-37.

11. Cover TM. Geometrical and Statistical Properties of Systems of Linear Inequalities with Applications in Pattern Recognition. IEEE Trans Electron Comput. 1965;3(EC-14):326–34.

World Medical Association. World Medical Association Declaration of Helsinki: Ethical Principles for Medical Research Involving Human Subjects. JAMA. November 27, 2013;310(20):2191-4.

13. Schuetze K, Eickhoff A, Rutetzki KS, Richter PH, Gebhard F, Ehrnthaller C. Geriatric patients with dementia show increased mortality and lack of functional recovery after hip fracture treated with hemiprosthesis. Eur J Trauma Emerg Surg Off Publ Eur Trauma Soc Jun 2022;48(3):1827-33.

14. Kocar Thomas, Denkinger M, Dallmeier D, Fotteler M, Leinert C. Evidence-based recommendations for acute orthogeriatric care: a systematic review of clinical practice guidelines. PROSPERO 2022 CRD42022292141 [Internet]. [cited August 12, 2022]; Available from: https://www.crd.york.ac.uk/prospero/display_record.php?ID=CRD42022292141

15. Leinert C, Fotteler M, Kocar T, Dhayana.Dallmeier, Denkinger M. Predictors and outcomes of interest of continuity of care decisions for older inpatients in acute care settings: a scoping review. March 24, 2022 [cited August 12, 2022]; Available from: https://osf.io/yjzax

16. Chisholm D, Knapp MR, Knudsen HC, Amaddeo F, Gaite L, van Wijngaarden B. Client Socio-Demographic and Service Receipt Inventory--European Version: development of an instrument for international research. EPSILON Study 5. European Psychiatric Services: Inputs Linked to Outcome Domains and Needs. Br J Psychiatry Suppl. 2000;(39):s28-33.

17. Roick C, Kilian R, Matschinger H, Bernert S, Mory C, Angermeyer MC. The German version of the Client Sociodemographic and Service Receipt Inventory. Psychiatr Prax. Oct 2001;28(Sup. 2):84-90.

18. Savvopoulos S, Sampalli T, Harding R, Blackmore G, Janes S, Kumanan K, et al. Development of a quality scoring tool to assess quality of discharge summaries. J Fam Med Prim Care. April 2018;7(2):394-400.

19. IHI Global Trigger Tool for Measuring Adverse Events (Second Edition) | IHI - Institute for Healthcare Improvement [Internet]. [cited August 1, 2022]. Available from: https://www.ihi.org/resources/Pages/IHIWhitePapers/IHIGlobalTriggerToolWhitePaper.aspx?PostAuthRed=/resources/_layouts/download.aspx?SourceURL=/resources/Knowledge%20Center%20Assets/IHIWhitePapers%20-%20IHIGlobalTriggerToolforMeasuringAdverseEventsSecondEdition_ab736cac-935b-46e4-b6fe-7f98c45f8a78/IHIGlobalTriggerToolWhitePaper2009.pdf

20. Hees VT van, Migueles JH, Sabia S, Patterson MR, Fang Z, Heywood J, et al. GGIR: Raw Accelerometer Data Analysis [Internet]. 2022 [cited January 11, 2023]. Available from: https://CRAN.R-project.org/package=GGIR

21. Olsen F, Lundborg F, Kristiansson J, Hård af Segerstad M, Ricksten SE, Nellgård B. Validation of the Nottingham Hip Fracture Score (NHFS) for the prediction of 30-day mortality in a Swedish cohort of hip fractures. Acta Anaesthesiol Scand. 2021;65(10):1413-20.

22. Eschweiler GW, Czornik M, Herrmann ML, Knauer YP, Forkavets O, von Arnim CAF, et al. Presurgical Screening Improves Risk Prediction for Delirium in Elective Surgery of Older Patients: The PAWEL RISK Study. Front Aging Neurosci [Internet]. 2021 [cited March 10, 2022];13. Available at: https://www.frontiersin.org/article/10.3389/fnagi.2021.679933

23 Kuhn-Thiel AM, Weiß C, Wehling M, FORTA authors/expert panel members. Consensus validation of the FORTA (Fit fOR The Aged) List: a clinical tool for increasing the appropriateness of pharmacotherapy in the elderly. Drugs Aging. February 2014;31(2):131-40.
