# Supplementary material for: Supporting SURgery with GEriatric Co-Management and AI (SURGE-Ahead): A study protocol for the development of a digital geriatrician
Source: PLoS One. 2023 Jun 16;18(6):e0287230. doi: 10.1371/journal.pone.0287230 (PMC10275448; doi:10.1371/journal.pone.0287230)
Supplement: S4 File — (PDF) [file pone.0287230.s004.pdf]

ENGLISH TRANSLATION

for the document titled

„Antrag Nr. 310/22 – SURGE-Ahead Observations- und KI-Entwicklungsstudie (OKIE)“

(For the original document in German please see supplement)

Application No. 310/22 - SURGE-Ahead Observation and AI Development Study

Dear Professor Denkinger,

Your above-mentioned application was submitted to the Ethics Committee of the University of Ulm for consultation on 05.09.2022. After receipt of the requested additions and corrections on 18.10.2022, all requirements are now fulfilled.

There are no ethically justifiable objections to the performance of the planned investigations.

This concludes the evaluation by the Ethics Committee of the University of Ulm with a favorable opinion.

In accordance with the exclusively advisory function of the Ethics Committee pursuant to § 15 of the Professional Code of Conduct of the Baden-Württemberg Medical Association, this evaluation concerns only the *professional ethical and legal assessment* of the conception, the planned methods, the implementation, and monitoring of the project in question, and the intended patient education.

The medical and legal responsibility remains unrestrictedly with the project manager and the participants.

- Recruitment of subjects/patients may only begin from the date of this vote.
- The Ethics Committee must be informed immediately of any serious or unexpected adverse events that occur during the study and could affect the safety of the study participants or the conduct of the project.
- Please notify us of any change in protocol performance. It must then be clarified whether the vote of the Ethics Committee of the University of Ulm is still valid.
- The Ethics Committee of the University of Ulm assumes that non-medical employees are specifically sworn to secrecy.
- We assume that all study-related additional costs will not be charged to the health insurance companies and that no official or other dependency relationships will be exploited in the recruitment of study participants.
- We would like to point out that, according to Art. 35 of the Declaration of Helsinki, clinical studies must be registered in a publicly accessible database (e.g., at the DRKS of the University of Freiburg) before the first subject is recruited.
- Data protection aspects of research projects are only examined cursorily by the ethics committee. This vote therefore does not replace the consultation of the responsible data protection officer.

- In addition, the ethics committee requires prompt notification of the completion of the study and a report with notification of the findings obtained.

We wish you every success in carrying out the study and ask you to send us the final report or the corresponding publication in due course.

For the Ethics Committee of the University of Ulm

Mrs. Prof. Dr. med. Manuela Dudeck
Deputy Chairperson

Attachment

The vote is based on the following documents:

Submitted documents [received 19.08.2022]:

- OKIE_Ethikantrag Ulm 17.08.2922, unterzeichnet durch Prof. Denkinger, Prof. Gebhard, Prof. Michalski, Prof. Bolenz, Prof. Kestler, Prof. Kilian, Prof. Steger, Frau PD DR. Dallmeier, Dr. Leinert, Frau Dr. Uihlein
- Begleit-Email Agaplesion Bethesda Klinik vom 18.08.2022 zur Antragseinreichung
- OKIE_Ausführliches Studienprotokoll August 2022
- OKIE_INFORMATION FÜR TEILNEHMENDE, Version Nr. 1, August 2022
- OKIE_INFORMATION FÜR GESETZLICHE VERTRETERINNEN UND VERTRETER, Version Nr. 1, August 2022
- OKIE_Einwilligungserklärung Teilnehmende, Version Nr. 1, August 2022
- OKIE_Einwilligung gesetzl. Vertreter:innen, Version Nr. 1, August 2022
- OKIE_Erhebungsbogen mit allen Assessments und Fragen, Version 1, August 2022
- OKIE_MASKE FÜR DIE DATENERFASSUNG, Version Nr. 1, August 2022
- OKIE_Identifikationsliste_Studienteilnehmende
- OKIE_SCHWEIGEPFLICHTENTBINDUNG FÜR ANGEHÖRIGE UND HAUSÄRZT:INNEN DER TEILNEHMER:INNEN
- OKIE_SCHWEIGEPFLICHTENTBINDUNG FÜR BEZUGSPERSONEN UND HAUSÄRZT:INNEN DER TEILNEHMER:INNEN
- OKIE_Screening-Liste
- OKIE_Verschwiegenheitserklärung, Version Nr. 1, August 2022
- OKIE_Datenblatt_AX6
- OKIE_Hygienekonzept, Version Nr. 1, August 2022

Subsequent documents [received 18.10.2022]:

- Antwort Agaplesion Bethesda Ulm vom 13.10.2022 zu den Nachforderungen
- OKIE_Studienprotokoll_Revision, Version 2 vom Oktober 2022
- OKIE_Übersicht Promotionen
- Information für gesetzliche Vetreter/innen, Version 2 vom Oktober 2022
- Teilnehmerinformation, Version 2 vom Oktober 2022
